# Drug Targets for Cell Cycle Dysregulators in Leukemogenesis: *In Silico* Docking Studies

**DOI:** 10.1371/journal.pone.0086310

**Published:** 2014-01-15

**Authors:** Archana Jayaraman, Kaiser Jamil

**Affiliations:** Centre for Biotechnology and Bioinformatics, School of Life Sciences, Jawaharlal Nehru Institute of Advanced Studies, Secunderabad, Andhra Pradesh, India; George Mason University, United States of America

## Abstract

Alterations in cell cycle regulating proteins are a key characteristic in neoplastic proliferation of lymphoblast cells in patients with Acute Lymphoblastic Leukemia (ALL). The aim of our study was to investigate whether the routinely administered ALL chemotherapeutic agents would be able to bind and inhibit the key deregulated cell cycle proteins such as - Cyclins E1, D1, D3, A1 and Cyclin Dependent Kinases (CDK) 2 and 6. We used Schrödinger Glide docking protocol to dock the chemotherapeutic drugs such as Doxorubicin and Daunorubicin and others which are not very common including Clofarabine, Nelarabine and Flavopiridol, to the crystal structures of these proteins. We observed that the drugs were able to bind and interact with cyclins E1 and A1 and CDKs 2 and 6 while their docking to cyclins D1 and D3 were not successful. This binding proved favorable to interact with the G1/S cell cycle phase proteins that were examined in this study and may lead to the interruption of the growth of leukemic cells. Our observations therefore suggest that these drugs could be explored for use as inhibitors for these cell cycle proteins. Further, we have also highlighted residues which could be important in the designing of pharmacophores against these cell cycle proteins. This is the first report in understanding the mechanism of action of the drugs targeting these cell cycle proteins in leukemia through the visualization of drug-target binding and molecular docking using computational methods.

## Introduction

Acute Lymphoblastic Leukemia (ALL) is characterized by uncontrolled proliferation of immature lymphoblast precursor cells. Based on the type of lymphoblast affected, ALL is of two types-T-ALL and B-ALL [1]. A number of genes and their products and genetic translocations have been reported to contribute to leukemogenesis [2]. Research studies show that many of these are involved either directly or indirectly in regulating the cell cycle and differentiation processes [3]. Deregulation of the cell cycle is one of the main deregulations in the transformation of a normal cell to a cancerous cell and hence the genes and proteins regulating this process serve as key therapeutic targets.

Cell division, which involves the cell cycle process, is essential in the production of new cells. The cell cycle process involves four phases –G1, S, G2 and Mitotic (M) phases. Each phase in itself and the transition from one phase to another is closely regulated by a series of molecules which include cyclins, cyclin dependent kinases and cyclin dependent kinase inhibitors. Deregulation of these molecules leads to aberrant processing and results in loss of control of normal cycling. Hence, these molecules serve as ideal targets to hinder abnormal cell proliferation [4].

**Figure 1:**
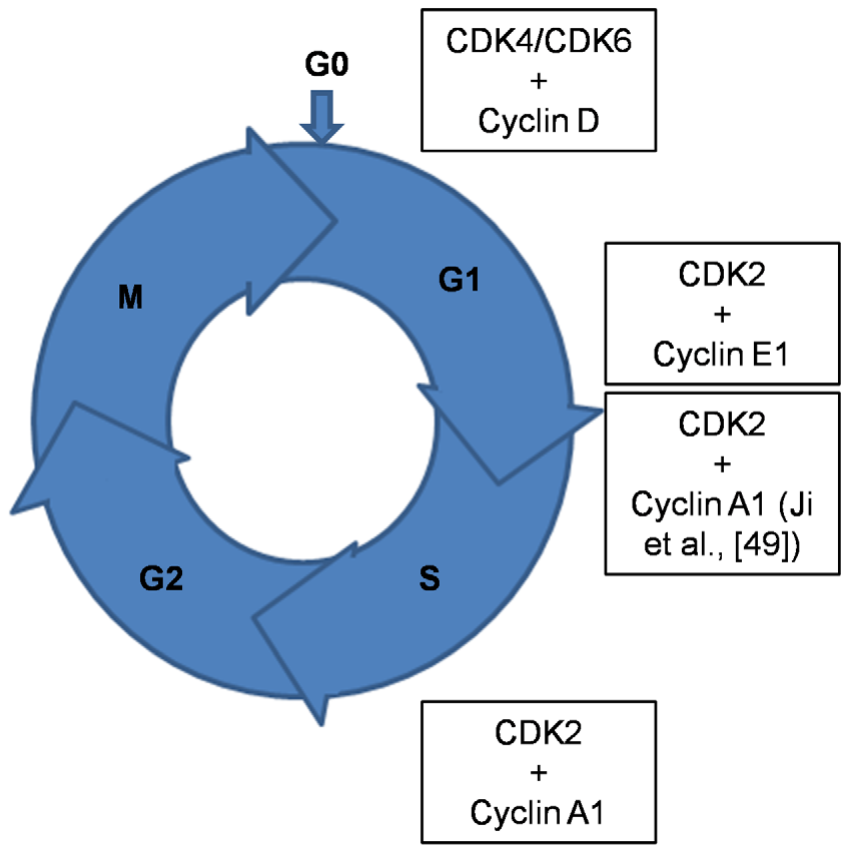
Cell cycle phases showing some of the check point proteins that can be deregulated in leukemia.

In ALL, alterations in Cyclins D1 -CCND1 [5], [6], D3 -CCND3 [7], E1 -CCNE1 [8] and A1-CCNA1 [9], Cyclin dependent kinases (CDK) 2 &6 -CDK2 & CDK6 [10], [11] [Figure 1], cyclin dependent kinase inhibitors CDKN2A, CDKN2B [12], CDKN1B [13], CDKN1C [14] have been reported. The differential expression, especially of the cyclins and the CDKs, leads to loss of checkpoint control and hence results in neoplastic transformation, which is also evident from our earlier studies [15]–[17] and hence these proteins would serve as important drug targets.

The tumor suppressor protein, Retinoblastoma (Rb), generally exists in complex with the E2F transcription factor family. The release of E2F from this complex initializes the transcription of genes which allow the transition from G1 to S phase. This release is mediated through the phosphorylation of the Rb protein by the cyclin D-CDK4/CDK6 and the cyclin E-CDK2 complexes [18]. Deregulated expression of the cyclin/CDK complexes causes an increase in the phosphorylation of Rb protein, leading to loss of check in the transition from G1/S phase and thus aberrant cell cycling occurs. Current treatment strategies targeting ALL involve the use of chemotherapeutic drugs such as Daunorubicin, Doxorubicin, Etoposide which target DNA topoisomerases while drugs such as Paclitaxel and Vindesine target the microtubules (http://www.cancer.gov/cancertopics/druginfo/ leukemia#dal1; www.drugbank.ca).

To understand the mechanism of action of the drug through the visualization of drug-target binding, molecular docking studies using computational methods proved helpful [19]. Our earlier studies have examined protein-drug interactions of several biomolecules involved in carcinogenesis [19]–[24]. In the current study, our aim was to examine whether the traditional chemotherapeutic agents administered for ALL interact with cell cycle proteins, and to compare their binding (score) with that of drugs specific to these cell cycle proteins. We also analysed the residues of the binding site and those involved in interaction with the ligands to determine their significance in the functioning of the protein and to explore their potential as targets in the design of more effective drugs.

## Methods

The crystal structures of the 6 proteins-CCNE1 (PDB ID: 1W98), CDK6 (PDB ID: 3NUP), CCND1 (PDB ID: 2W96), CCND3 (PDB ID: 3G33), CDK2 (PDB ID: 1W98) and CCNA1 (PDB ID: 1FIN) were retrieved from RCSB Protein Data Bank (PDB) (www.rcsb.org/pdb/home/home.do). They were imported into the Schrödinger Maestro suite 2012 (Schrödinger, LLC, New York, NY, 2012) for preparation, minimization and docking studies. The Maestro suite is a comprehensive collection of software programs that are useful in biomolecule structure visualization, protein homology modeling, docking and pharmacophore design. It incorporates both a command-line interface and a graphical user interface, allowing the users greater control over the parameters and execution of the various applications.

**Figure 2:**
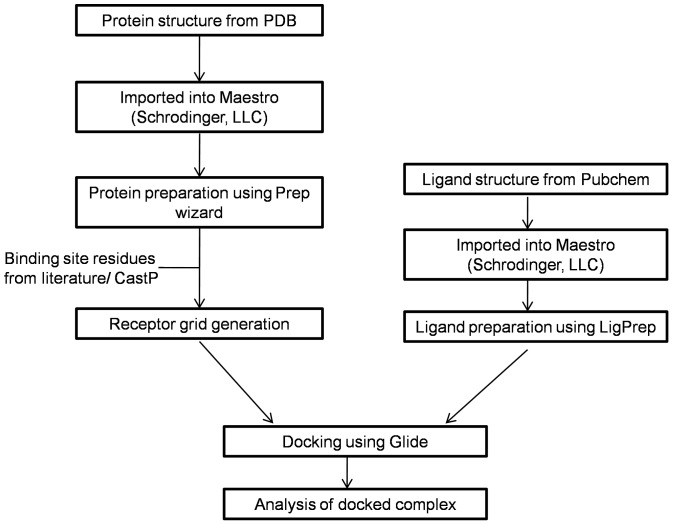
Flowchart of the methodology followed while docking target protein to ligand.

The residue numbering throughout the manuscript and figures is according to the PDB crystal structure of each protein. The flowchart in Figure 2 depicts the sequence of steps followed in the docking of each of the protein targets to the ligands investigated in our study.

The list of drugs generally used for the treatment of ALL patients was retrieved from the National Cancer Institute's list of drugs approved for Acute Lymphoblastic Leukemia (http://www.cancer.gov/cancertopics/druginfo/leukemia#dal1). Information about the proteins that they generally target is available in DrugBank (http://www.drugbank.ca/; [25]), which is a database with comprehensive information about drugs, their activity and their targets. Of these, the drugs involved in cell cycle arrest were chosen as the target proteins are involved in regulating cell cycle. 6-Mercaptopurine was also included, as it is also part of the treatment strategy for ALL [26]. Teniposide was also included as it is administered to ALL patients with induction failure [27], [28]. cis-Resveratrol [29], Flavopiridol [30], PD0332991 [31], 3,3′-Diindolylmethane [32] and 5,7-Dimethoxyflavone [33] have also been observed to have toxic effects on leukemic cells in studies on ALL samples. The drugs Indirubin [34], AZD5438 [35], Bryostatin-1 [36] and CCT020312 [37], which have been reported to inhibit cell proliferation, were also tested against the cell cycle proteins investigated in our study.

To investigate the significance of the docking of the chemotherapeutic drugs, administered for ALL, to the proteins, we compared their binding (score) to that of the drugs reported to regulate the expression of these proteins. These drugs were retrieved from BindingDB (Binding database, http://www.bindingdb.org/bind/index.jsp, [38]), which is a repository containing information on the binding and bioactivity of drugs and their targets. The database has been compiled from information based on experimental published reports. The information on the drugs can be retrieved from the database through a search using the protein name as query or through browsing the list of the available targets. The references for these specific drugs are available in File S1.

The structures of the ligands for both chemotherapeutic and specific drugs were retrieved from PubChem (http://pubchem.ncbi.nlm.nih.gov/) database through a query using the compound name and were prepared and minimized prior to docking. We used 29 ligands for CCNE1, 29 ligands for CDK6, 28 ligands for CCND3, 34 ligands for CCND1, 32 ligands for CDK2 and 20 ligands for CCNA1 in our docking studies (Table S1 in File S1).

### Protein Preparation

The crystal structures of the proteins were prepared prior to docking using the Protein Preparation wizard (Schrödinger Suite 2012 Protein Preparation Wizard, Impact version 5.8, Prime version 3.1, Schrödinger, LLC, New York, NY, 2012) in Maestro (Maestro, version 9.3, Schrödinger, LLC, New York, NY, 2012). Since the crystal pdb structures of the protein may have problems in the structure data such as improper bond orders, missing side chains, etc, they need to be prepared using the Protein Preparation wizard prior to docking. This process checks the protein structure to verify proper assignment of bonds and bond orders, to add hydrogens, to detect disulphide bonds, to fill in any missing loops or side chains and to correct any mislabeled elements. Further, the protein structure was subjected to restrained minimization in the Impref utility using the OPLS2005 force field. During this minimization process the heavy atoms in the structure are restrained to relieve torsional strain with a harmonic potential of 25 kcal mol^−1^ Å^−2^ and hydrogens remain unrestrained. We retained the default number of four iterations for refinement. Protein Structure Preparation does not alter the geometry of the input protein; instead it checks for any problems in the protein structure and corrects them.

### Ligand Preparation

The ligand structures downloaded from PubChem were prepared for docking using LigPrep (LigPrep, version 2.5, Schrödinger, LLC, New York, NY, 2012) application in Maestro. LigPrep ensures optimization of ligand geometry, removes ligands with structural problems and generates structural variants. LigPrep generated low energy 3D structure with optimized chiralities for the input ligands (Schrodinger LigPrep Manual). We generated different ionization states of the ligands at pH 7.0+/−2.0 using Epik version 2.3 (Schrödinger, LLC, New York, NY, 2012; [39]) and the ligand was minimized using OPLS2005 force field.

### Docking

#### Receptor Grid Generation

The receptor grid is the three dimensional boundary for the binding of ligands. The receptor grid was created using Receptor grid generation in the Glide application [40] (Glide, version 5.8, Schrödinger, LLC, New York, NY, 2012) of Maestro (Schrödinger, LLC, New York, NY, 2012). The receptor grid for the proteins in this study was generated by specifying the binding (active) site residues, which were extracted from literature relevant to each protein. CastP server [41] was used for CDK6 and CCND3 to obtain additional information on their binding site residues. Uniprot database (www.uniprot.org) was also queried to obtain information regarding domains that may be important for protein function.

#### Active site residues

The binding site residues for CCNE1 were retrieved from the study on the crystal structure of CCNE1 by Honda et al. [42]. These residues have been observed to contribute to the structural and functional properties of the protein. The binding site residues for CDK6 were retrieved from the interactions with the ligand crystallized in the pdb structure [43], from the sequence annotation information in Uniprot database (Accession number: Q00534) and from the probable binding site predicted by CastP server having the highest site volume (827.2 Å^3^). The binding site residues predicted by CastP (site volume = 373.3 Å^3^) for the CCND3 protein were used for grid generation. The binding site residues for CCND1 were retrieved from the study by Day et al. [44] on the crystal structure of the protein in complex with CDK4 (Cell cycle Dependent Kinase 4) protein. The binding site residues for CDK2 were retrieved from the studies on the protein crystal structure by Clare et al. [45], Honda et al. [42] and Betzi et al. [46]. The binding site residues for CCNA1, used for grid generation, were retrieved from the study by Jeffrey et al. [47] on the crystal structure of CCNA1 and the residues that play an important role in the structure and functioning of the protein. The binding site residues for each protein are available in Table 1. These residues are important as they may influence ligand entry and binding of the ligand to the protein. These residues may also play a crucial role in functioning of the protein via interactions with residues of other biomolecules that bind to these proteins.

**Table 1:** Binding site residues for grid generation for each of the target proteins.

S.No.	Target Protein Name	Binding site residues-receptor grid generation
1.	CCNE1	Lys 108, Tyr 112, Arg 130, Lys 186, Leu 187, Glu 188, Leu 195, Glu 215, Lys 220, Asn 236, Tyr 243, Leu 244, Asn 245, Asp 246, Leu 247, His 248, Glu 249, Leu 251
2.	CDK6	Ile 19, Gly 20, Gly 25, Lys 26, Val 27, Ala 41, Lys 43, Leu 65, Leu 68, Glu 69, Glu 72, Val 76, Val 77, Arg 78, Leu 79, Leu 96, Phe 98, Glu 99, His 100, Val 101, Asp 102, Gln 103, Asp 104, Phe 135, Asp 145, Lys 147, Gln 149, Asn 150, Leu 152, Ala 162, Asp 163, Phe 164, Leu 166
3.	CCND3	Tyr 38, Val 39, Pro 40, Arg 57, Pro 79, Met 82, Asn 83, Asp 86, Val 155, Ile 156, Ala 157, His 158, Leu 186, Cys 189, Ala 190, Phe 195, Ala 196, Tyr 198, Pro 200, Ile 203
4.	CCND1	Thr 37, Met 56, Ile 59, Trp 63, Glu 66, Arg 87, Lys 96, Gln 100, Lys 112, Ala 121, Asp 129, Glu 141, Asn 151
5.	CDK2	Tyr 15, Lys 33, Ile 35, Leu 37, Glu 42, Val 44, Arg 50, Ile 52, Leu 55, Lys 56, Glu 57, Val 64, His 71, Leu 76, Leu 78, Phe 80, Phe 82, His 84, Asp 86, Asp 145, Phe 146, Arg 150, Val 154, Arg 157
6.	CCNA1	Tyr 178, Ile 182, Tyr 185, Gln 228, Lys 266, Phe 267, Glu 269, Ile 270, Glu 295, Thr 303, Phe 304, Asp 305

#### Glide docking

Once the receptor grid is generated, the ligands are docked to the receptor using Glide version 5.8 (Grid based LIgand Docking with Energetics) docking protocol [40]. The ligands were docked using “xtra precision mode” (XP). During docking, the protein was rigid while the ligands were flexible. Glide generates different conformations internally and these are passed through a set of filters namely euler angles, grid-based force field evaluation and refinement and Monte Carlo energy minimization. Finally, the docked conformers are evaluated using Glide (G) Score and a single best pose per ligand is generated as output. The GScore is calculated as follows:
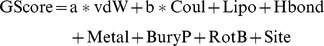

wherein vdW denotes van der Waals energy, Coul denotes Coulomb energy, Lipo denotes lipophilic contact, HBond indicates hydrogen-bonding, Metal indicates metal-binding, BuryP indicates penalty for buried polar groups, RotB indicates penalty for freezing rotatable bonds, Site denotes polar interactions in the active site and the a = 0.065 and b = 0.130 are coefficients of vdW and Coul

The Glide score is an empirical scoring function that is an approximation of the ligand binding free energy and incorporates many parameters such as force fields and penalties for interactions that influence ligand binding as stated by Schrodinger knowledge base (http://www.schrodinger.com/kb/1027). It has also been stated to “have been optimized for docking accuracy, database enrichment and binding affinity prediction” (http://www.schrodinger.com/kb/1027). It is expressed in the units- kcal/mol (http://www.schrodinger.com/kb/1015).

#### Statistical analysis

All the statistical analysis in this investigation have been performed by Maestro software (Schrödinger Suite, LLC, New York, NY, 2012) and presented in various tables as docking scores and bond distances. The docking studies were repeated atleast twice to check the replication of the results obtained. For the target proteins which were used for ligand docking, we checked the poses with minimum energy score and highest binding affinity.

#### Docking using decoys from *Directory of Useful Decoys* (DUD) database

To further validate our results, we queried the Directory of Useful Decoys (DUD) database (http://dud.docking.org/ [48]) which contains a set of drug-like molecules that have similar physical properties and dissimilar topology to ligands that bind to a set of specific protein target molecules. These decoys serve as a benchmark in evaluating the results obtained from the various docking software that are available. Of the six proteins analysed in our study, decoys (n = 2074) were available only for CDK2 protein and were used for docking with the ligands in our study after ligand preparation. The decoy molecules were downloaded in sdf format (http://dud.docking.org/r2/databases/dud_decoys2006/cdk2_decoys.sdf.gz).

## Results and Discussion

Leukemia treatment regimens involve targeting cancer cells for destruction. The chemotherapeutic drugs bind to their target molecules, inhibiting them, thus preventing their function. In our study, we evaluated the ability of the routinely administered ALL chemotherapeutic drugs to bind and inhibit cell cycle proteins that are overexpressed in leukemic cells. For each protein, the docked poses were evaluated and the pose with the highest docking score (a more negative value), followed by highest gscore (a more negative value) and lowest docking energy were selected.

### CCNE1

Scuderi et al. [8] reported that overexpression of cyclin E1 in ALL cells may be related to advanced stage of disease. Thus, chemotherapeutic agents that bind to the protein can help reduce levels of cyclin E1 and prevent progression of cell cycle from G1 to S phase. Analysis of the docked ligands in our study showed that the glide score range was between −8.978264 kcal/mol and −2.867899 kcal/mol (Table 2). We observed that Doxorubicin and its less toxic analog Daunorubicin were able to bind to the cyclin E1 protein with the highest docking gscore of −8.978264 kcal/mol and −7.76946 kcal/mol respectively. In comparison to the known cyclin E1 inhibitors and compounds observed to downregulate cyclin E1 expression such as Baicalein and Silymarin, the common ALL chemotherapeutic agents seem to have an equivalent or better glide GScore. Baicalein and Silymarin were observed to bind to the protein with a gscore of −6.693247 kcal/mol and −5.573164 kcal/mol. The phytoconstituents Berberine and Curcumin were observed to have a gscore of −4.47667 kcal/mol and −4.461087 kcal/mol respectively. The other chemotherapeutic agents in our docking study which were observed to have favorable binding include Teniposide, Flavopiridol, Vindesine and Nelarabine.

**Table 2:** CCNE1 Glide docking scores.

S.No.	PubChem id	Entry Name	Docking Score (kcal/mol)	XP GScore	Glide Gscore (kcal/mol)	Glide Emodel (kcal/mol)
1.	31703	Doxorubicin	−8.95156	−8.97826	−8.97826	−68.999329
2.	30323	Daunorubicin	−7.74926	−7.76946	−7.76946	−64.196671
3.	5281605	Baicalein	−6.67385	−6.69325	−6.69325	−36.410978
4.	34698	Teniposide	−6.63204	−6.63204	−6.63204	−70.258213
5.	5330286	PD0332991	−4.55541	−6.29041	−6.29041	−59.792509
6.	5287969	Flavopiridol	−6.13114	−6.13354	−6.13354	−44.767621
7.	40839	Vindesine	−5.99959	−6.09019	−6.09019	−63.483014
8.	CCT020312	CCT020312	−5.58332	−5.60432	−5.60432	−66.633883
9.	1548994	Silymarin	−5.57316	−5.57316	−5.57316	−45.485382
10.	5281607	Chrysin	−5.42404	−5.44024	−5.44024	−33.895111
11.	54454	Simvastatin	−5.33656	−5.33656	−5.33656	−47.360709
12.	16747683	AZD5438	−5.02819	−5.27199	−5.27199	−49.244388
13.	248862	Nelarabine	−4.90822	−4.90822	−4.90822	−42.895914
14.	3071	3,3′-diindolylmethane	−4.90367	−4.90367	−4.90367	−33.509082
15.	126941	Methotrexate	−4.83814	−4.86284	−4.86284	−59.239153
16.	88881	5,7-dimethoxyflavone	−4.58961	−4.58961	−4.58961	−25.125536
17.	2353	Berberine	−4.47667	−4.47667	−4.47667	−45.917302
18.	969516	Curcumin	−4.46109	−4.46109	−4.46109	−50.567365
19.	5359405	Indirubin	−4.41973	−4.42573	−4.42573	−37.941561
20.	119182	Clofarabine	−4.33962	−4.33962	−4.33962	−33.046098
21.	1548910	cis-Resveratrol	−4.28236	−4.28236	−4.28236	−29.749806
22.	92729	gamma-Tocopherol	−4.00713	−4.00713	−4.00713	−41.172173
23.	5154	Sanguinarine	−3.87291	−3.87291	−3.87291	−31.941554
24.	5359405	Staurosporine	−3.62298	−3.68068	−3.68068	−51.452803
25.	25235992	Bryostatin-1	−3.54027	−3.54027	−3.54027	−55.709117
26.	370	Gallic Acid	−3.21306	−3.21606	−3.21606	−30.077516
27.	36314	Paclitaxel	−3.10523	−3.10523	−3.10523	−85.260876
28.	5144	Safrole	−3.05067	−3.05067	−3.05067	−20.055171
29.	667490	6-Mercaptopurine	−2.2625	−2.8679	−2.8679	−26.045568

**Figure 3:**
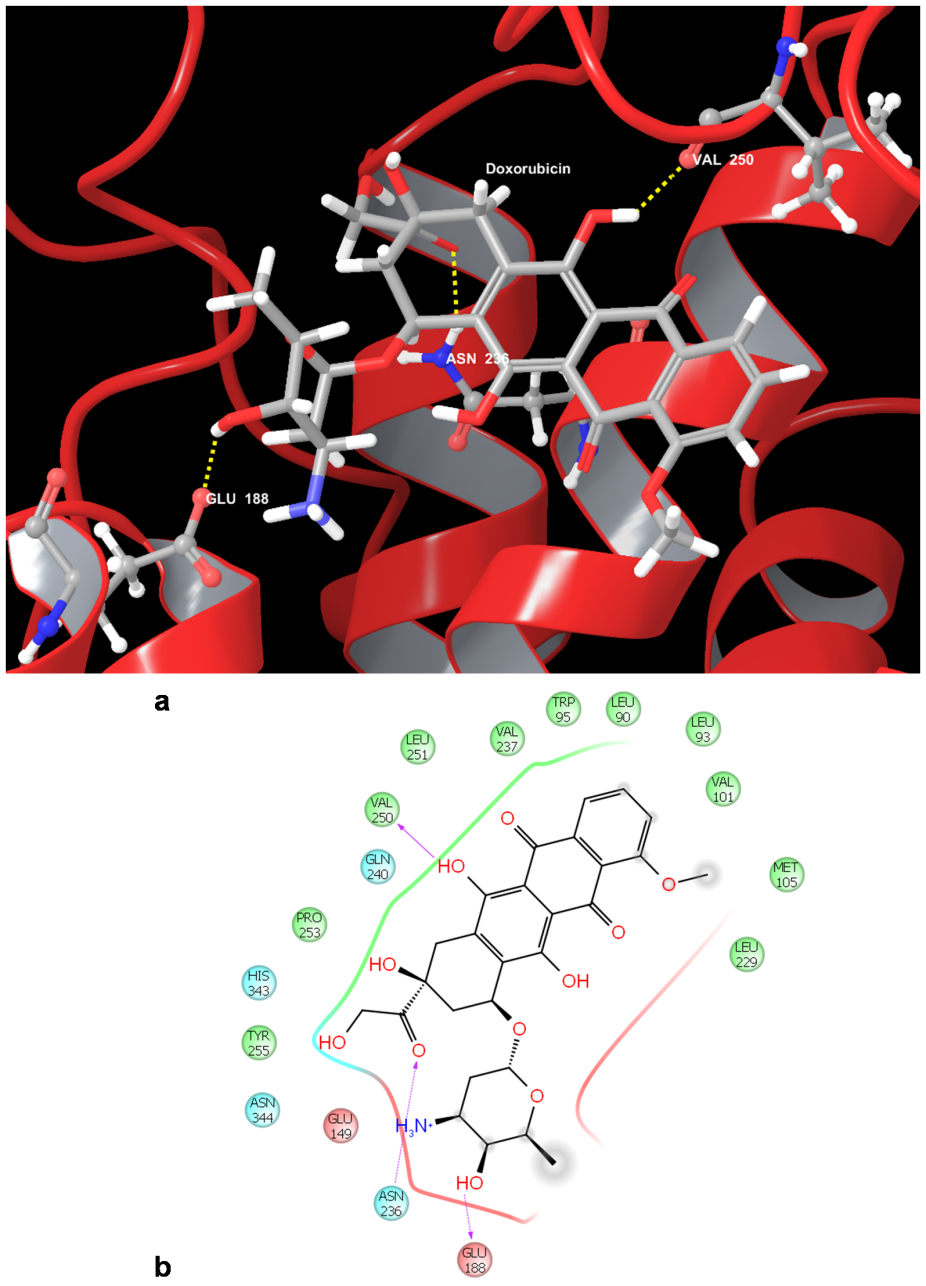
Docked pose of Cyclin E1 (CCNE1) with Doxorubicin. a. Structural view wherein hydrogen bonding is shown as yellow dashed line. b. Ligand interaction diagram with pink arrows representing electrostatic interactions and green line represent π-π interactions.

Of the 18 residues identified through literature [42] as important binding site residues, 13 were polar and 5 were hydrophobic. The top scoring ligands Doxorubicin (Figure 3) and Daunorubicin were observed to interact with two residues – Glu 188 and Asn 236 through Hydrogen bonding with their side chains; in addition they were also observed to form Hydrogen bond with the backbone of Val 250. Asn 236 was observed to be an important residue interacting with many of the ligands/drugs.

Honda et al. [42] reported that Glu 188 is an important residue which is involved in interactions with CDK2. The residue Asn 236 was reported by Matsumoto and Maller [49] as one of the residues that is involved in binding to centrosomes and aids in DNA synthesis independent of CDK2. These residues have been suggested to play a role in CDK2-dependent regulation of cell cycle transition and CDK2-independent involvement in DNA synthesis by Cyclin E1 [42]. We also observed that some of the ligands interact with Leu 251. This residue was reported by Honda et al [38] to form hydrogen bond with Val156 of CDK2 and van der Waals interaction with Phe 152 and Val 156 of CDK2. Interaction of the drugs with Leu 251 could hinder bond formation with the residues of CDK2, thus preventing CCNE1/CDK2 complex formation and hence affect their functioning. The binding of the ALL chemotherapeutic drugs Doxorubicin and Daunorubicin to these residues would make them unavailable to interact with CDK2 and may thus serve in inhibiting CCNE1-CDK2 complex formation.

The other residues that were observed to interact with the ligands were Trp 95, Lys 108, Tyr 255, Asp 341, Ser 233, Pro 228, Asn 344, Ser 227. Of the 12 residues interacting with the various docked ligands, 8 were polar and 4 were hydrophobic. We also observed that, of all the residues analysed, the residues that are involved in interactions with most of the ligands are Trp 95, Asn 236, Val 250, Leu 251, Glu 188. These may serve as an important consideration in drug design due to their ability to interact with the many ligands.

Honda et al. [42] have also reported that mutation of Ser 233 to Ala would affect cyclin E binding to centrosome and may also alter conformation. The drugs analysed in our study did not interact with this residue with a high frequency and hence mutation of this residue may not affect binding of these drugs to the CCNE1 protein.

These observations suggest that a combination of ALL therapeutic agents with the phytoconstituents could be more effective in binding to cyclin E1, preventing its interaction with CDK2, thus inhibiting proliferation of leukemic cells mediated by this protein.

### CDK6

Increased expression of *CDK6* in ALL and its role in pathogenesis was reported by Chilosi et al [11]. Agirre et al. [50] reported that abnormal proliferation in ALL cells due to increased expression of *CDK6* is mediated by *hsa-miR-124a* miRNA and they suggested that inhibition of the CDK6 protein may serve as an important therapeutic strategy for ALL. Thirty three residues were identified from crystal structure [43] and from Uniprot and CastP server as important binding site residues as they are either involved in interactions with the ligand or are present in the active site of the protein and may thus be involved in influencing ligand entry into binding cavity. Of these, 18 were hydrophobic and 15 were polar residues.

Of the input binding site residues, the residues Ile 19, Gly 25, Lys 43, Phe 98, Glu 99, His 100, Val 101, Asp 102, Asp 104, Asp 145, Lys 147, Gln 149, Asp 163 were found to interact with the ligands used for the docking study. Of the 16 residues observed to interact with the ligand, 12 were polar and 4 were hydrophobic. The residues Val 101, Lys 43, Glu 99, Gly 25, His 100, Asp 104, Gln 149, Asp 163 were observed to have the most number of interactions with the ligands.

The residues Lys 43 and Val 101 were observed to interact with the ligand in the crystal structure [43] implicating these residues as important binding site residues. In our docking analysis also, we observed many of the ligands interacting with these residues through Hydrogen bonds, thus emphasizing their importance as targets for inhibitors.

**Figure 4:**
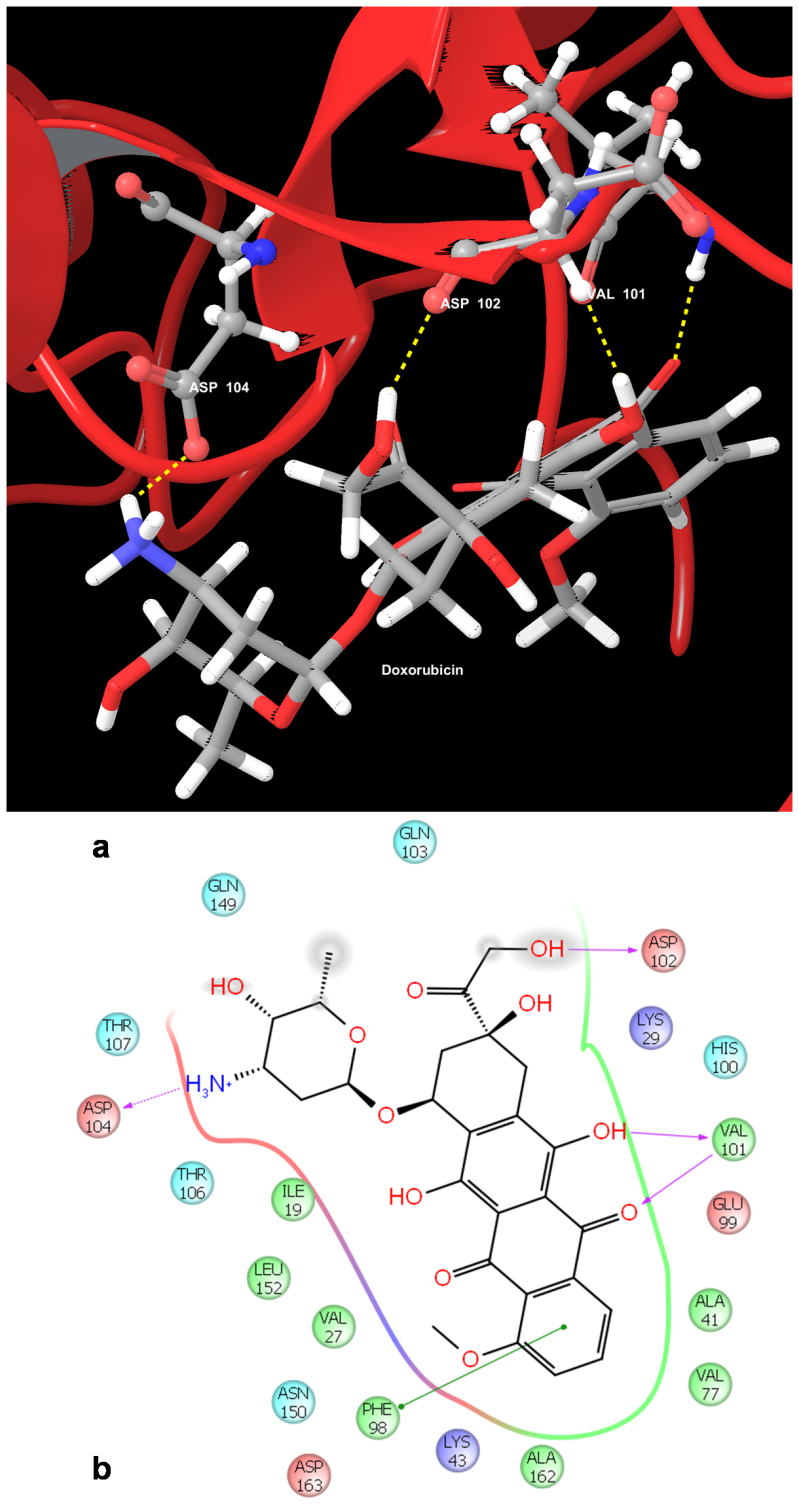
Docked pose of Cyclin Dependent Kinase 6 (CDK6) with Doxorubicin. a. Structural view wherein hydrogen bonding is shown as yellow dashed line. b. Ligand interaction diagram with pink arrows representing electrostatic interactions and green line represent π-π interactions.

CHEMBL1230169 and Fisetin, which have been reported to reduce the levels of CDK6, were observed to have a gscore of −9.97081 kcal/mol and −8.66307 kcal/mol respectively. The chemotherapeutic drugs Doxorubicin (Figure 4) and Daunorubicin were observed to bind to CDK6 protein with higher docking gscores (−9.15692 kcal/mol and −8.70732 kcal/mol respectively) than the other ALL drugs (Table 3). The plant products Curcumin and Flavopiridol were observed to have a gscore of −8.58057 kcal/mol and −8.32896 kcal/mol respectively.

**Table 3:** CDK6 Glide docking scores.

S.No.	PubChem id	Entry Name	Docking Score (kcal/mol)	XP GScore	Glide Gscore (kcal/mol)	Glide Emodel (kcal/mol)
		3NU	−9.70384	−10.117	−10.117	−53.9526
1.	49800099	CHEMBL1230169	−9.55761	−9.97081	−9.97081	−52.5371
2.	31703	Doxorubicin	−9.13022	−9.15692	−9.15692	−64.3092
3.	5359405	Indirubin	−9.07878	−9.08478	−9.08478	−46.1933
4.	30323	Daunorubicin	−8.68712	−8.70732	−8.70732	−66.4128
5.	5281614	Fisetin	−8.65707	−8.66307	−8.66307	−50.941
6.	969516	Curcumin	−8.58057	−8.58057	−8.58057	−62.6672
7.	44202892	Saikosaponin A	−8.41152	−8.41152	−8.41152	−43.3885
8.	16747683	AZD5438	−8.09967	−8.34347	−8.34347	−59.3648
9.	5287969	Flavopiridol	−8.32656	−8.32896	−8.32896	−58.4897
10.	3071	3,3′-diindolylmethane	−7.80221	−7.80221	−7.80221	−46.5999
11.	5330286	PD0332991	−7.74647	−7.80197	−7.80197	−76.5415
12.	5280443	Apigenin	−7.26546	−7.28226	−7.28226	−60.5444
13.	5280442	Acacetin	−7.20046	−7.21566	−7.21566	−57.9883
14.	31553	Silibinin	−7.21253	−7.21253	−7.21253	−60.2264
15.	119182	Clofarabine	−7.15835	−7.15835	−7.15835	−47.9438
16.	5281607	Chrysin	−6.96403	−6.98023	−6.98023	−52.3565
17.	9549304	Aminopurvalanol	−6.69247	−6.69247	−6.69247	−68.5812
18.	1548910	cis-Resveratrol	−6.65205	−6.65205	−6.65205	−45.6071
19.	CCT020312	CCT020312	−6.42486	−6.44586	−6.44586	−75.1481
20.	667490	6-Mercaptopurine	−5.58344	−6.18884	−6.18884	−38.2607
21.	88881	5,7-dimethoxyflavone	−5.98475	−5.98475	−5.98475	−41.5574
22.	442126	Decursin	−5.40202	−5.40202	−5.40202	−26.6856
23.	248862	Nelarabine	−5.28558	−5.28558	−5.28558	−51.0729
24.	36314	Paclitaxel	−4.77711	−4.77711	−4.77711	−71.303
25.	126941	Methotrexate	−4.67404	−4.69874	−4.69874	−62.96
26.	40839	Vindesine	−3.59592	−3.68652	−3.68652	−22.112
27.	16046126	CHEMBL215803	−3.05478	−3.26828	−3.26828	−36.1927
28.	34698	Teniposide	−3.23853	−3.23853	−3.23853	−50.7281

Also, to test the docking efficiency, we docked the crystal structure ligand 3NU along with the other ligands. The interactions in the crystal structure were replicated through our docking study.

### CCND3

Cyclin D3 is an important cell cycle gene that is expressed specifically in certain tissues such as lymphoid and endocrine tissues. This gene has been reported to play an important role in proliferation and in initiation and maintenance of differentiation of cells [51]. Several studies have reported abnormal expression of cyclin D3 in ALL and have suggested its association with increase in proliferation of immature T lymphocytes [7], [52].

Twenty residues were reported by CastP server and Uniprot to be important for binding. Of these, 7 were polar and 13 were hydrophobic. The major residues that were observed to interact with the ligands were Asn 83, Asp 86, Ala 157, Tyr 38, Tyr 198, Leu 186, Arg 57; 5 of these are polar and 2 were hydrophobic. Asp 86 and Ala 157 were observed to interact with more number of ligands.

**Figure 5:**
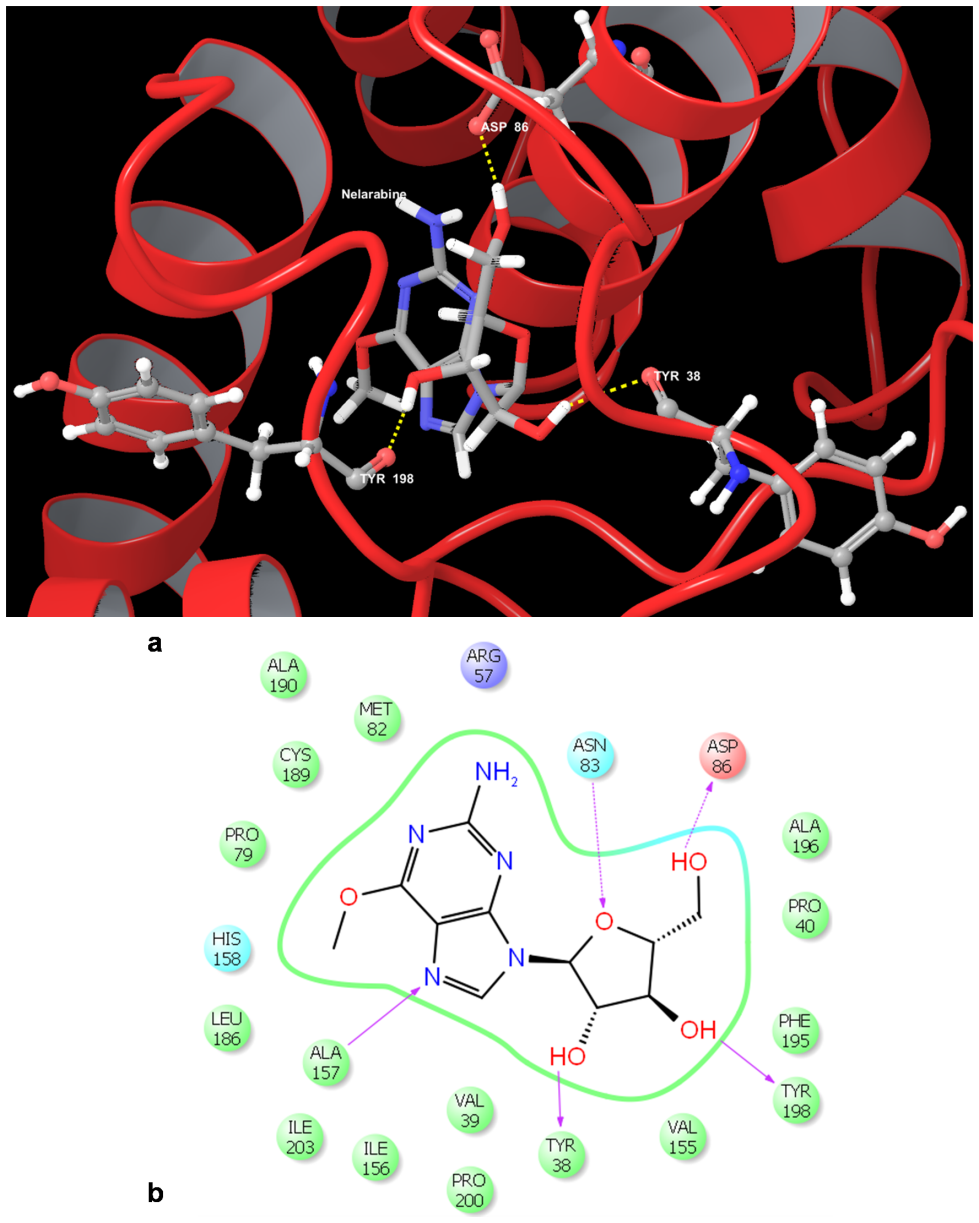
Docked pose of Cyclin D3 (CCND3) with Nelarabine. a. Structural view wherein hydrogen bonding is shown as yellow dashed line. b. Ligand interaction diagram with pink arrows representing electrostatic interactions and green line represent π-π interactions.

Although twenty eight ligands were used in our docking study, we observed that only eight ligands had docked to the receptor; which suggests that many of the ALL chemotherapeutic agents may not be effective drugs against this protein. Of the bound ligands, the ligand with the highest gscore was the chemotherapeutic drug Nelarabine (−7.492605 kcal/mol) (Table 4) (Figure 5). This drug was observed to interact with the protein forming five hydrogen bonds with the residues Asn 83, Asp 86 (side chain interactions), Ala 157, Tyr 38, Tyr 198 (backbone interactions). The ligand with the second highest gscore was observed to be Clofarabine (−6.548855 kcal/mol) which formed two hydrogen bonds with the side chain of Asp 86 and backbone of Leu 186 amino acids. These results suggest that Nelarabine could be an effective drug against cyclin D3.

**Table 4:** CCND3 Glide docking scores.

S.No.	PubChem id	Entry Name	Docking Score (kcal/mol)	XP GScore	Glide Gscore (kcal/mol)	Glide Emodel (kcal/mol)
1.	248862	Nelarabine	−7.49261	−7.49261	−7.49261	−20.6297
2.	119182	Clofarabine	−6.54886	−6.54886	−6.54886	−26.5355
3.	5281607	Chrysin	−5.23431	−5.25051	−5.25051	18.55504
4.	667490	6-Mercaptopurine	−4.50248	−5.96738	−5.96738	−37.1435
5.	5359405	Indirubin	−4.29363	−4.29963	−4.29963	22.65299
6.	1548910	cis-Resveratrol	−3.42338	−3.42338	−3.42338	−21.6323
7.	3071	3,3′-diindolylmethane	−2.36215	−2.36215	−2.36215	12.61512
8.	88881	5,7-dimethoxyflavone	−0.36688	−0.36688	−0.36688	34.29739

### CCND1

Cyclin D1 is an important regulator of G1/S transition in cell cycle. Volm et al. [5] implicated increased expression of cyclin D1 as a prognostic factor in childhood ALL. Thirty four ligands were tested for docking against Cyclin D1 receptor. Of these, we observed that only nine ligands were able to bind to the receptor.

Thirteen residues were input from the published literature [44] as residues important for binding. Of these, 9 were polar and 4 were hydrophobic. The residues interacting with the ligands were observed to be Trp 63, Lys 96 and Gln 100. Other than these residues, Ser 131 was also found to form Hydrogen bond with the ligand Curcumin. Of these 4 residues which are involved in interactions with the ligand, 3 were polar and 1 was hydrophobic. Trp 63 and Lys 96 were observed to interact the most with the ligands.

**Figure 6:**
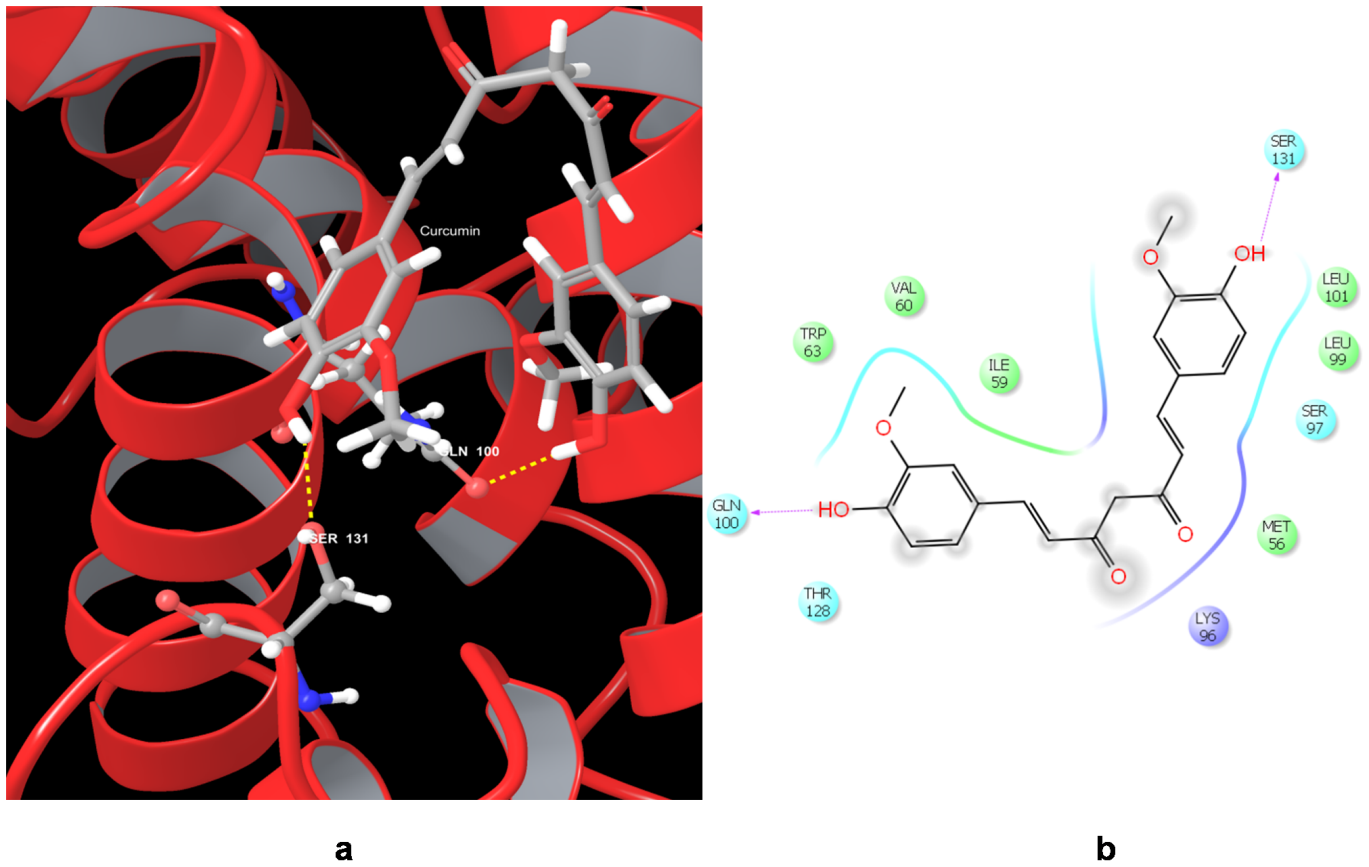
Docked pose of Cyclin D1 (CCND1) with Curcumin. a. Structural view wherein hydrogen bonding is shown as yellow dashed line. b. Ligand interaction diagram with pink arrows representing electrostatic interactions and green line represent π-π interactions.

In our study, we observed that the ALL chemotherapeutic drugs did not bind favorably with the cyclin D1 protein. Only the drug 3, 3′-diindolylmethane was observed to interact with the protein through a Π-Π interaction with the residue Trp 63 and very low docking gscore of −1.977454 kcal/mol (Table 5). In comparison, compounds such as the phytoconstituents Curcumin (Figure 6) and Baicalein, which are known to reduce cyclin D1 expression, were observed to have a better gscore of −3.366368 kcal/mol and −2.42012 kcal/mol respectively. These observations indicate that the current ALL chemotherapeutic drugs may not be effective in controlling proliferation through inhibition of cyclin D1.

**Table 5:** CCND1 Glide docking scores.

S.No.	Pubchem id	Entry Name	Docking Score (kcal/mol)	XP GScore	Glide Gscore (kcal/mol)	Glide Emodel (kcal/mol)
1.	969516	Curcumin	−3.36637	−3.36637	−3.36637	−37.6403
2.	5281605	Baicalein	−2.40072	−2.42012	−2.42012	−28.3858
3.	5281426	7-hydroxycoumarin	−2.26202	−2.26852	−2.26852	−21.5461
4.	456214	Purvalanol A	−2.0064	−2.0064	−2.0064	−36.321
5.	3071	3,3′-diindolylmethane	−1.97745	−1.97745	−1.97745	−29.1947
6.	5327723	Arcyriaflavin A	−1.91133	−1.91133	−1.91133	0
7.	3278	Ethacrynic Acid	−1.6757	−1.6757	−1.6757	−28.1984
8.	5359405	Indirubin	−0.15866	−0.16466	−0.16466	−25.1833
9.	9797847	Imide Analog 12	0.393207	0.393207	0.393207	−29.1047

### CDK2

CDK2 is an important cell cycle regulatory protein which forms a complex with cyclin E which is involved in phosphorylation of Retinoblastoma tumor suppressor protein along with cyclin D/CDK 4,6 complex. Phosphorylation by CDK2 reduces the affinity of Rb to remain attached to the nuclear matrix and thus ensures removal of this protein from nucleus by buffers [10]. Schmitz et al. [10] reported the presence of catalytic activity of CDK2 in childhood ALL samples and suggested that this activity may contribute to Rb inactivation in leukemic cells. Thirty two ligands were used in the docking study against CDK2 and we observed 30 ligands to bind to the receptor.

Twenty four residues which were obtained through literature search [42], [45], [46] were used as input as binding site residues. These residues are involved in inducing phosphorylation and some residues (Asp 145, Phe 146, Arg 150, Val 154, Arg 157) are also part of the activation site [42]. Of the twenty four residues, 12 were polar and 12 were hydrophobic residues. The residues that were observed to interact with the ligand were Tyr 15, Lys 33, Asp 86, Asp 145; other than these the residues Ile 10, Glu 12, Thr 14, Gly 16, Ser 46, Thr 47, Glu 51, Leu 83, Asp 127, Lys 129, Gln 131, Asn 132, Glu 162 were also observed to interact with the ligand through Hydrogen bonds, Π-cation and Π-Π interactions. Of these 17 interacting residues, 3 were hydrophobic and 14 were polar residues. The residues, Lys 33, Tyr 15, Thr 14, Thr 47, Glu 51, Leu 83, Lys 129, Asn 132, Asp 145, were found to interact the most with the docked ligands.

Honda et al. [42] observed that Asp 145 is part of the activation segment of CDK2, which plays a role in substrate recognition and Lys 33 may be involved in stabilizing the triphosphates moiety of ATP for catalysis. Betzi et al. [46] observed that Tyr 15 is involved in interaction with the extrinsic fluorophore 8-anilino-1-naphthalene sulfonate (ANS). Many of the drugs used in our docking study were found to interact with either or all of these three residues highlighting the significance of these residues for the design of CDK2 inhibitors.

**Figure 7:**
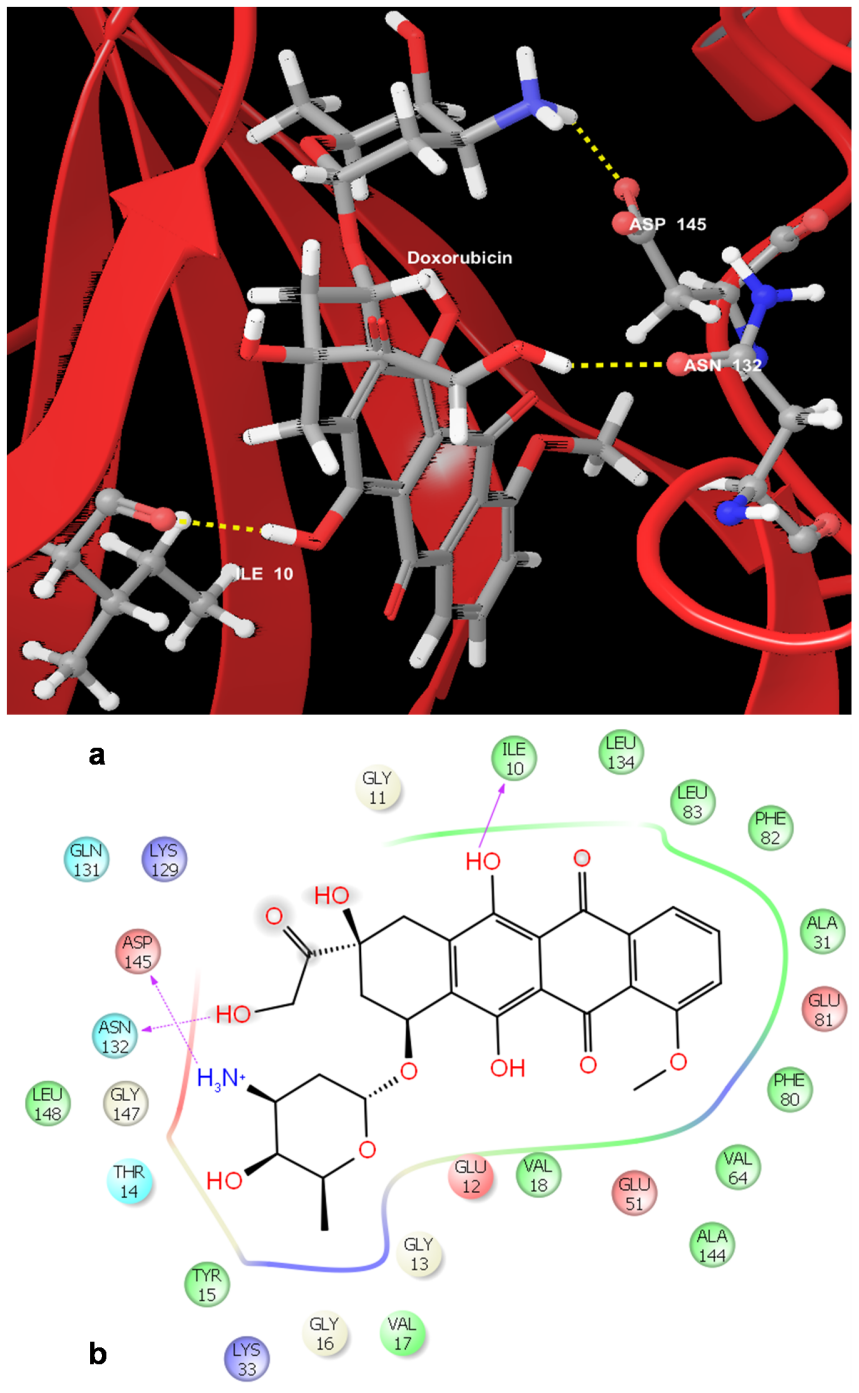
Docked pose of Cyclin Dependent Kinase 2 (CDK2) with Doxorubicin. a. Structural view wherein hydrogen bonding is shown as yellow dashed line. b. Ligand interaction diagram with pink arrows representing electrostatic interactions and green line represent π-π interactions.

The docking results (Table 6) from our study showed that the chemotherapeutic agents, Doxorubicin (Figure 7) and Daunorubicin, bind to CDK2 with high gscores of −10.125607 kcal/mol and −9.392828 kcal/mol respectively. Most of the other chemotherapeutic agents were also observed to bind favorably to CDK2 suggesting that these drugs could serve as effective inhibitors of this protein. The phytoconstituents such as Flavopiridol [53] and Curcumin and Quercetin were also observed to bind favorably with CDK2 with gscores of −7.239727 kcal/mol and −6.962194 kcal/mol and −6.28335 kcal/mol. The ligands used routinely as CDK2 inhibitors were observed to have gscores in the range of −8.030843 kcal/mol to −3.48897 kcal/mol.

**Table 6:** CDK2 Glide docking scores.

S.No.	PubChem id	Entry Name	Docking Score (kcal/mol)	XP GScore	Glide Gscore (kcal/mol)	Glide Emodel (kcal/mol)
1.	31703	Doxorubicin	−10.0989	−10.1256	−10.1256	−92.8855
2.	30323	Daunorubicin	−9.37263	−9.39283	−9.39283	−77.6861
3.	5281800	Acteoside	−8.03084	−8.03084	−8.03084	−73.7168
4.	5287969	Flavopiridol	−7.23733	−7.23973	−7.23973	−63.1122
5.	969516	Curcumin	−6.96219	−6.96219	−6.96219	−44.7846
6.	5280343	Quercetin	−6.27625	−6.28335	−6.28335	−51.2667
7.	119182	Clofarabine	−5.72474	−5.72474	−5.72474	−45.4839
8.	25125014	CHEMBL1234833	−5.68257	−5.68257	−5.68257	−52.0839
9.	46926350	SCH727965_dinaciclib	−5.29195	−5.29195	−5.29195	−54.2394
10.	1548910	cis-Resveratrol	−5.22929	−5.22929	−5.22929	−45.7782
11.	160355	Roscovitine	−5.15061	−5.18051	−5.18051	−53.7752
12.	248862	Nelarabine	−5.05059	−5.05059	−5.05059	−45.7547
13.	11285002	RGB 286638	−4.00761	−5.03151	−5.03151	−80.8429
14.	5330286	PD0332991	−4.94583	−5.00133	−5.00133	−71.8033
15.	126941	Methotrexate	−4.96596	−4.99066	−4.99066	−70.1398
16.	CCT020312	CCT020312	−4.87839	−4.89939	−4.89939	−59.8627
17.	5359405	Indirubin	−4.88691	−4.89291	−4.89291	−41.3814
18.	16747683	AZD5438	−4.57746	−4.82126	−4.82126	−51.4876
19.	3071	3,3′-diindolylmethane	−4.62176	−4.62176	−4.62176	−45.865
20.	34698	Teniposide	−4.43071	−4.43071	−4.43071	−44.7312
21.	5281607	Chrysin	−4.32732	−4.34352	−4.34352	−38.8003
22.	60138160	4-[(E)-(6-hydroxy-2-oxo-1,2-dihydro pyridin- 3-yl)diazenyl] benzene sulfonamide	−4.19366	−4.19366	−4.19366	−53.3649
23.	53249966	CHEMBL1800452	−4.00454	−4.17384	−4.17384	−44.1582
24.	23727981	Meriolin 3	−4.02848	−4.03778	−4.03778	−45.2406
25.	2608	U55	−3.8204	−3.9039	−3.9039	−45.6715
26.	88881	5,7-dimethoxyflavone	−3.86511	−3.86511	−3.86511	−33.9364
27.	40839	Vindesine	−3.52796	−3.61856	−3.61856	−40.7999
28.	9817550	Variolin B	−3.44877	−3.48897	−3.48897	−49.3641
29.	667490	6-Mercaptopurine	−2.82366	−3.42906	−3.42906	−35.3069
30.	36314	Paclitaxel	−2.44526	−2.44526	−2.44526	−36.7989

#### Docking using decoys from *Directory of Useful Decoys* (DUD) database

We have performed docking with the decoys from DUD database for CDK2. Our results showed Doxorubicin as the top scoring ligand with a gscore of −10.126 kcal/mol. This result is similar to our original docking results, obtained through docking without the use of decoys from DUD database. Also, the docking scores obtained for the top scoring ligands in our original docking results were also similar to the docking results (Daunorubicin = −9.393 kcal/mol; Acetoside = −8.031 kcal/mol; Flavopiridol = −7.240 kcal/mol; Curcumin = −6.962 kcal/mol) using decoys. These results add to the significance of our docking study. A few of the decoys such as ZINC01133013 (−9.555 kcal/mol), ZINC01133011 (−9.546 kcal/mol) scored better than some of the drugs evaluated in our study. The significance of these molecules needs to be explored further.

### CCNA1

Cyclin A1 has been reported to be expressed in hematopoietic progenitor cells. Typically, CCNA1/CDK2 functions at S/G2 phase. However Ji et al. [54] have observed that in somatic cells, cyclin A1 may contribute to G1/S transition in cell cycle and that increased expression of cyclin A1 augments entry into S phase and thus contributes to neoplastic progression. Holm et al. [9] reported overexpression of Cyclin A1 in Childhood ALL samples and they associated this increased expression with poor event-free survival. Thus, cyclin A1 protein serves as a viable target against leukemogenesis.

Twenty ligands were tested for docking against Cyclin A1 receptor and we observed 18 to bind favorably to the receptor. From the study by Jeffrey et al. [47], twelve residues were identified as important for binding; of these 8 were polar and 4 were hydrophobic residues. We observed that the residues Lys 266, Phe 267, Glu 269, Ile 270, Phe 304 interact with many of the ligands through Hydrogen bonds and Π-Π interactions. Of the 14 interacting residues, 9 were polar and 5 were hydrophobic.

The residues Lys 266, Phe 267, Ile 270 and Phe 304, which were identified as binding site residues from the crystal structure, have been reported to form part of the cyclin box region of the cyclin A1 protein [47]. The interaction between the cyclin box region of this proteins and the PSTAIRE region of CDK2 has been suggested to form an interface when binding to CDK2 and confers specificity for binding of cyclin A to CDK2 [47]. The drug-residue interactions observed in our study thus prevent formation of interface, hinder binding of cyclin A1 to CDK2 and prevent complex formation. Thus, these drugs may be explored for treatment in leukemic patients with cyclin A1 overexpression, especially as they bind to Leu 266, Phe 267 and Phe 304. These residues have been reported by Jeffrey et al. [47] to interact with residues of CDK2 facilitating CCNA/CDK2 complex formation. The binding of the drugs analysed in our study to these residues may disrupt this complex formation by making them unavailable to interact with the residues of CDK2. The other residues that were found to interact with the ligand include Asn 173, Glu 174, Asn 229, Glu 230, Leu 263, Glu 268, Ala 307, Asn 312 and Gln 313. Of these, the residues Asn 229, Glu 230, Leu 263, Glu 268 are also part of the cyclin box and may serve as drug targets in preventing binding of CDK2 to cyclin A.

**Figure 8:**
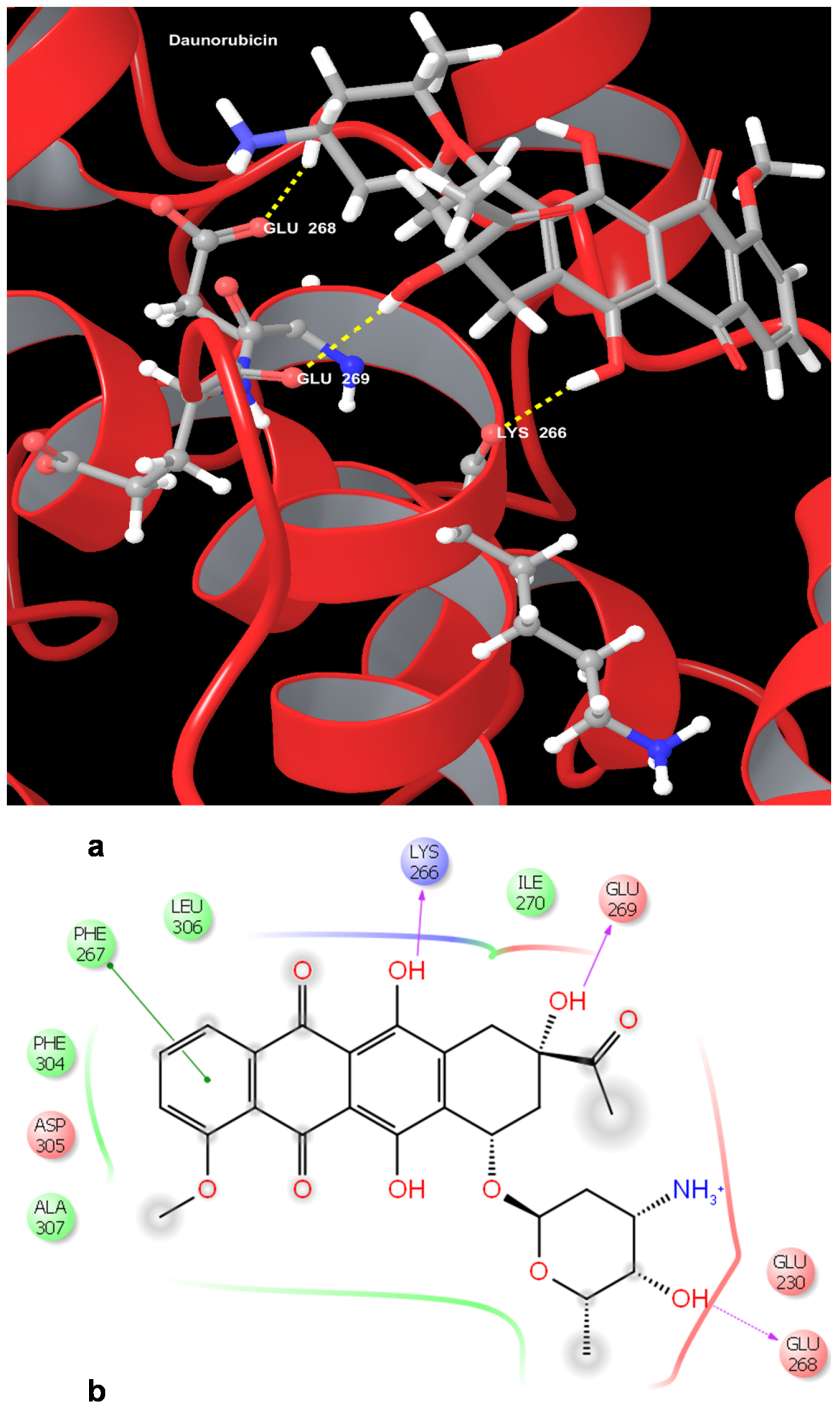
Docked pose of Cyclin A1 (CCNA1) with Daunorubicin. a. Structural view wherein hydrogen bonding is shown as yellow dashed line. b. Ligand interaction diagram with pink arrows representing electrostatic interactions and green line represent π-π interactions.

Our analysis of the docking results (Table 7) showed that the chemotherapeutic agents Daunorubicin (Figure 8) and Vindesine were found to have the highest gscores of −5.791 kcal/mol and −5.35824 kcal/mol. The phytoconstituents Flavopiridol and Curcumin were also found to bind with gscores of −4.932635 kcal/mol and −3.396278 kcal/mol respectively.

**Table 7:** CCNA1 Glide docking scores.

S.No.	PubChem id	Entry Name	Docking Score (kcal/mol)	XP GScore	Glide Gscore (kcal/mol)	Glide Emodel (kcal/mol)
1.	30323	Daunorubicin	−5.7708	−5.791	−5.791	−54.2684
2.	40839	Vindesine	−5.26764	−5.35824	−5.35824	−49.1995
3.	5330286	PD0332991	−5.05873	−5.11423	−5.11423	−48.0631
4.	5287969	Flavopiridol	−4.93024	−4.93264	−4.93264	−25.2734
5.	31703	Doxorubicin	−4.75327	−4.77997	−4.77997	−63.6425
6.	160355	R-Roscovitine	−4.60244	−4.63234	−4.63234	−35.4638
7.	36314	Paclitaxel	−4.40485	−4.40485	−4.40485	−74.651
8.	1548910	cis-Resveratrol	−4.11108	−4.11108	−4.11108	−25.6718
9.	3071	3,3′-diindolylmethane	−4.09934	−4.09934	−4.09934	−32.9399
10.	119182	Clofarabine	−3.6552	−3.6552	−3.6552	−36.3254
11.	88881	5,7-dimethoxyflavone	−3.40699	−3.40699	−3.40699	−27.278
12.	969516	Curcumin	−3.39628	−3.39628	−3.39628	−36.7028
13.	248862	Nelarabine	−3.34326	−3.34326	−3.34326	−33.5086
14.	126941	Methotrexate	−3.28501	−3.30971	−3.30971	−44.4237
15.	CCT020312	CCT020312	−3.13115	−3.15215	−3.15215	−45.3803
16.	16747683	AZD5438	−2.80621	−3.05001	−3.05001	−41.203
17.	667490	6-Mercaptopurine	−2.07168	−2.67708	−2.67708	−20.1023
18.	5359405	Indirubin	−2.2963	−2.3023	−2.3023	−30.0724

Information about the Hydrogen bonding interaction and bond distances of all the target proteins and their docked ligands are given in Table S2 in File S1.

### Analysis of binding site residues and ligand interacting residues of the target proteins

We analysed the residue properties of the binding site and the residues interacting with the ligand in the four cyclin and two cyclin dependent kinase target proteins to understand the nature of the residues that may contribute to the functioning of the protein. Also, the properties of these residues could play an important role in the design of ligand through identification of R-groups capable of interacting with these residues.

#### Cyclin box region

In our study we observed that, of the residues interacting with the ligand, one residue (Glu 188) in CCNE1, three residues (Arg 57, Asn 83, Asp 86) in CCND3, four residues (Trp 63, Lys 96, Gln 100, Ser 131) in CCND1 and nine residues (Asn 229, Glu 230, Leu 263, Lys 266, Phe 267, Glu 268, Glu 269, Ile 270) in CCNA1 were part of the cyclin box region. In the cyclin proteins, the cyclin box region has been reported to play an important role in binding and interactions with CDK proteins [55]. This region generally spans about 100 residues and is important for the functioning of the protein due to their interactions with the amino acids of CDKs facilitating cyclin-CDK complex formation. Designing ligands that bind to these residues could block these residues from interacting with other proteins and may thus hinder the binding interactions of the cyclin proteins. The cyclin box residues involved in interactions with the ligands, in our study, may thus help prevent the binding of the cyclins with the CDKs, inhibiting their functioning in phase transition of cell cycle. The inhibition of this binding could consequently serve in curtailing neoplasticity of the leukemic cells.

We also observed a high frequency of occurrence of polar residues at the input binding sites and the observed interacting sites. Polar residues have been reported to form energy hot spots [56] and to be a part of regions involved in protein interactions [57]. In the cyclin proteins under study, the high density of polar residues at the binding site suggests that these contribute to their binding to CDKs and the subsequent phosphorylation of Rb mediated by the cyclin-CDK complex. These polar binding site and interacting residues may hence constitute important targets in drug design.

Comparison of input binding site residues among the four cyclin proteins revealed that the most frequently occurring amino acids were: His, Glu, Lys, Asn, Tyr, Arg, Asp, Glu and Thr (Polar), Val, Leu, Met, Phe and Ala (Hydrophobic). Comparative analysis of the observed interacting residues among the four cyclins showed that the residues most frequently involved in interactions were: Asn, Ser, Glu, Asp, Tyr, Gln and Lys (Polar) and Leu, Trp and Ala (Hydrophobic).

#### Comparison of residues between CDK6 and CDK2

Comparison of the input binding site residues between CDK6 and CDK2 showed that the residues that occurred more frequently were: Asp, Lys, Glu, Arg and His (polar); Leu, Val, Phe and Ile (Hydrophobic). The polar residues Asp, Gln, Lys, Glu and Thr and the hydrophobic residues Ile and Gly were observed to be involved in more number of interactions with the ligand in both the CDKs. This is the first report which has identified the binding site residues for all the ligands studied to the cell cycle proteins investigated in our analysis.

#### Comparison of binding of ALL chemotherapeutic drugs among the four cyclins and between cyclins and CDKs

The chemotherapeutic drugs, Doxorubicin and Daunorubicin, were observed to have a higher glide gscore in cyclin E1 when compared with cyclin A1 (cyclin D1 and D3 were not compared as these drugs did not dock to both these proteins) (Tables 2 and 7). Comparison between CDK2 and CDK6 showed that the two drugs had a slightly higher glide gscore in CDK2 (Tables 3 and 6). Both of the drugs had a higher gscore in CDKs than in cyclins which could indicate that they may be more effective in inhibition of cyclin dependent protein kinases than cyclins.

Since the cyclins and CDKs are deregulated in other cancers, these drugs could be tested in the other cancer cell lines also to examine their efficacy in inhibiting these proteins in these neoplasms. Also, since the cyclins and the cyclin dependent kinases are involved in a cascade of reactions, inhibition of these proteins may be useful in regulating their role in the carcinogenesis process.

Our study has shown that polar residues occur at the binding site with high frequency and play an important role in the binding interactions of the protein. These polar residues, along with the hydrophobic residues that interact with the ligand, may be important for the functional interactions of the protein, especially in protein-protein interactions. Therefore, drugs that are able to bind to these residues could serve as potent inhibitors of these proteins. Also, the residues surrounding the ligand are more or less equally distributed between polar and hydrophobic residues indicating the need for ligands with functional groups that are able to interact with both these amino acid groups to increase binding affinity and hence therapeutic efficacy.

In our docking study, we have evaluated twelve chemotherapeutic ligands for their binding ability to cell cycle proteins. We observed that the chemotherapeutic drugs Doxorubicin and Daunorubicin and several others such as Clofarabine, Nelarabine and Flavopiridol were able to interact favorably with the G1/S cell cycle proteins investigated and may lead to their inhibition in leukemic cells. Our results suggest that these drugs may also be effective as inhibitors of cell cycle proteins in conjunction with their current use as inhibitors of topoisomerases and DNA polymerases, thus extending their functionality. We have elucidated for the first time that the many of the chemotherapeutic drugs interact favourably when they are within the polar and hydrophobic residues of the target protein's grids.

The advantages of using Schrödinger software suite for docking studies lies in the degree of sensitivity and the ease of visualization of binding of the ligands to the protein active site. We could identify the crucial amino acids involved in interactions with the ligand and the nature of these interactions with the help of the software suite. The use of such *in silico* docking programs helps us understand the mechanism of drug binding to the protein target through a virtual simulation of the binding and in the generation of properties associated with binding.

Our study demonstrates for the first time the binding interactions of the cell cycle proteins and the chemotherapeutic ligands used for docking; and it has also identified residues that may serve as an important consideration in the design of drugs/pharmacophores to more effectively target the cell cycle proteins. In the light of these new findings, we propose that new ligands could also be generated or analogues of existing drugs could be easily evaluated using these new targets for control of tumorogenesis. This is the first report of the chemotherapeutic ligand binding to the new targets; the drugs which have shown a good binding score could be more effective in controlling cell cycle progression in leukemic cells.

## Supporting Information

File S1 Table S1, Ligands used for docking. Table S2, Hydrogen bond distances.
(DOC)
