# Supplementary material for: Drug Targets for Cell Cycle Dysregulators in Leukemogenesis: In Silico Docking Studies
Source: PLoS One. 2014 Jan 15;9(1):e86310. doi: 10.1371/journal.pone.0086310 (PMC3893288; doi:10.1371/journal.pone.0086310)
Supplement: File S1 — Table S1, Ligands used for docking. Table S2, Hydrogen bond distances. (DOC) [file pone.0086310.s001.doc]

**File S1**

**Drug Targets for Cell Cycle dysregulators in Leukemogenesis: *in silico* docking studies**

Archana Jayaraman^1^, Kaiser Jamil^1,*^

^1^Centre for Biotechnology and Bioinformatics, School of Life Sciences, Jawaharlal Nehru Institute of Advanced Studies, Secunderabad, Andhra Pradesh, India

**Supporting Information**

**Table S1. Ligands used for docking***

| **S.No.** | **PubChem id** | **Drug Name** | **Reference** |
| --- | --- | --- | --- |
| **CCNE1** |  |  |  |
| 1. | 25235992 | Bryostatin-1 | [1] |
| 2. | CCT020312 | CCT020312 | [2] |
| 3. | 3071 | 3,3'-diindolylmethane | [3] |
| 4. | 30323 | Daunorubicin |  |
| 5. | 31703 | Doxorubicin |  |
| 6. | 34698 | Teniposide | [4, 5] |
| 7. | 5281607 | Chrysin | [6] |
| 8. | 5287969 | Flavopiridol | [7] |
| 9. | 5330286 | PD0332991 | [8] |
| 10. | 5359405 | Indirubin | [9] |
| 11. | 16747683 | AZD5438 | [10] |
| 12. | 88881 | 5,7-dimethoxyflavone | [6] |
| 13. | 119182 | Clofarabine |  |
| 14. | 126941 | Methotrexate |  |
| 15. | 248862 | Nelarabine |  |
| 16. | 667490 | 6-Mercaptopurine | [11] |
| 17. | 969516 | Curcumin | [12] |
| 18. | 1548910 | cis-Resveratrol | [13] |
| 19. | 370 | Gallic Acid | [14] |
| 20. | 2353 | Berberine | [15] |
| 21. | 5144 | Safrole | [16] |
| 22. | 5154 | Sanguinarine | [17] |
| 23. | 44259 | Staurosporine | [18,19] |
| 24. | 54454 | Simvastatin | [20] |
| 25. | 92729 | gamma-Tocopherol | [21] |
| 26. | 1548994 | Silymarin | [22] |
| 27. | 5281605 | Baicalein | [23] |
| 28. | 36314 | Paclitaxel |  |
| 29. | 40839 | Vindesine |  |
| **CDK6** |  |  |  |
| 1. | 25235992 | Bryostatin-1 | [1] |
| 2. | CCT020312 | CCT020312 | [2] |
| 3. | 3071 | 3,3'-diindolylmethane | [3] |
| 4. | 30323 | Daunorubicin |  |
| 5. | 31703 | Doxorubicin |  |
| 6. | 34698 | Teniposide | [4,5] |
| 7. | 5287969 | Flavopiridol | [7] |
| 8. | 5330286 | PD0332991 | [8] |
| 9. | 5359405 | Indirubin | [9] |
| 10. | 16747683 | AZD5438 | [10] |
| 11. | 5281607 | Chrysin | [6] |
| 12. | 88881 | 5,7-dimethoxyflavone | [6] |
| 13. | 119182 | Clofarabine |  |
| 14. | 126941 | Methotrexate |  |
| 15. | 248862 | Nelarabine |  |
| 16. | 667490 | 6-Mercaptopurine | [11] |
| 17. | 969516 | Curcumin | [12] |
| 18. | 1548910 | cis-Resveratrol | [13] |
| 19. | 442126 | Decursin | [23] |
| 20. | 5280442 | Acacetin | [24] |
| 21. | 5280443 | Apigenin | [25] |
| 22. | 5281614 | Fisetin | [25, 26] |
| 23. | 9549304 | Aminopurvalanol | [27] |
| 24. | 16046126 | CHEMBL215803 | [28] |
| 25. | 44202892 | Saikosaponin A | [29] |
| 26. | 49800099 | CHEMBL1230169 | [30] |
| 27. | 31553 | Silibinin | [31] |
| 28. | 36314 | Paclitaxel |  |
| 29. | 40839 | Vindesine |  |
| **CCND3** |  |  |  |
| 1. | CCT020312 | CCT020312 | [2] |
| 2. | 3071 | 3,3'-diindolylmethane | [3] |
| 3. | 30323 | Daunorubicin |  |
| 4. | 31703 | Doxorubicin |  |
| 5. | 34698 | Teniposide | [4, 5] |
| 6. | 88881 | 5,7-dimethoxyflavone | [6] |
| 7. | 119182 | Clofarabine |  |
| 8. | 126941 | Methotrexate |  |
| 9. | 248862 | Nelarabine |  |
| 10. | 667490 | 6-Mercaptopurine | [11] |
| 11. | 969516 | Curcumin | [12] |
| 12. | 1548910 | cis-Resveratrol | [13] |
| 13. | 5281607 | Chrysin | [6] |
| 14. | 5287969 | Flavopiridol | [7] |
| 15. | 5330286 | PD0332991 | [8] |
| 16. | 5359405 | Indirubin | [9] |
| 17. | 16747683 | AZD5438 | [10] |
| 18. | 25235992 | Bryostatin-1 | [1] |
| 19. | 2913 | Cyproheptadine | [32] |
| 20. | 64971 | Betulinic acid | [33] |
| 21. | 2732983 | S14161 | [34] |
| 22. | 5281800 | Acteoside | [35] |
| 23. | 11977753 | NVP BEZ235 | [36] |
| 24. | 16211710 | Silibinin | [31] |
| 25. | 44295234 | Rapamycin | [37] |
| 26. | 5280343 | Quercetin | [38] |
| 27. | 36314 | Paclitaxel |  |
| 28. | 40839 | Vindesine |  |
| **CCND1** |  |  |  |
| 1. | CCT020312 | CCT020312 | [2] |
| 2. | 3071 | 3,3'-diindolylmethane | [3] |
| 3. | 30323 | Daunorubicin |  |
| 4. | 31703 | Doxorubicin |  |
| 5. | 34698 | Teniposide | [4,5] |
| 6. | 88881 | 5,7-dimethoxyflavone | [6] |
| 7. | 119182 | Clofarabine |  |
| 8. | 126941 | Methotrexate |  |
| 9. | 248862 | Nelarabine |  |
| 10. | 667490 | 6-Mercaptopurine | [11] |
| 11. | 969516 | Curcumin | [12] |
| 12. | 1548910 | cis-Resveratrol | [13] |
| 13. | 5281607 | Chrysin | [6] |
| 14. | 5287969 | Flavopiridol | [7] |
| 15. | 5330286 | PD0332991 | [8] |
| 16. | 5359405 | Indirubin | [9] |
| 17. | 16747683 | AZD5438 | [10] |
| 18. | 25235992 | Bryostatin-1 | [1] |
| 19. | 3278 | Ethacrynic Acid | [39] |
| 20. | 36314 | Paclitaxel |  |
| 21. | 36462 | Etoposide | [40] |
| 22. | 44259 | Staurosporine | [18,19] |
| 23. | 369954 | 5,3'-dihydroxy-3,6,7,8,4'-pentamethoxyflavone | [41] |
| 24. | 456214 | Purvalanol A | [42] |
| 25. | 1548994 | Silymarin | [22] |
| 26. | 2732983 | S14161 | [34] |
| 27. | 5281426 | 7-hydroxycoumarin | [43] |
| 28. | 5281605 | Baicalein | [22] |
| 29. | 5281708 | Daidzein | [44] |
| 30. | 5327723 | Arcyriaflavin A | [45] |
| 31. | 5330286 | PD0332991 | [8] |
| 32. | 9797847 | Imide Analog 12 | [45] |
| 33. | 541 | Vincristine |  |
| 34. | 40839 | Vindesine |  |
| **CDK2** |  |  |  |
| 1. | 46926350 | SCH727965_dinaciclib | [46] |
| 2. | CCT020312 | CCT020312 | [2] |
| 3. | 3071 | 3,3'-diindolylmethane | [3] |
| 4. | 30323 | Daunorubicin |  |
| 5. | 31703 | Doxorubicin |  |
| 6. | 34698 | Teniposide | [4,5] |
| 7. | 88881 | 5,7-dimethoxyflavone | [6] |
| 8. | 119182 | Clofarabine |  |
| 9. | 126941 | Methotrexate |  |
| 10. | 248862 | Nelarabine |  |
| 11. | 667490 | 6-Mercaptopurine | [11] |
| 12. | 969516 | Curcumin | [12] |
| 13. | 1548910 | cis-Resveratrol | [13] |
| 14. | 5281607 | Chrysin | [6] |
| 15. | 5287969 | Flavopiridol | [7] |
| 16. | 5330286 | PD0332991 | [8] |
| 17. | 5359405 | Indirubin | [9] |
| 18. | 16747683 | AZD5438 | [10] |
| 19. | 25235992 | Bryostatin-1 | [1] |
| 20. | 2608 | U55 | [47] |
| 21. | 9817550 | Variolin B | [48] |
| 22. | 23727981 | Meriolin 3 | [49] |
| 23. | 53249966 | CHEMBL1800452 | [50] |
| 24. | 60138160 | 4-[(E)-(6-hydroxy-2-oxo-1,2-dihydropyridin- 3-yl)diazenyl] benzenesulfonamide | [51] |
| 25. | 5281800 | Acteoside | [35] |
| 26. | 5280343 | Quercetin | [38] |
| 27. | 160355 | Roscovitine | [52] |
| 28. | 25125014 | CHEMBL1234833 | [53] |
| 29. | 11285002 | RGB 286638 | [54] |
| 30 | 72271 | 7-hydroxystaurosporine | [55] |
| 31. | 36314 | Paclitaxel |  |
| 32. | 40839 | Vindesine |  |
| **CCNA1** |  |  |  |
| 1. | 25235992 | Bryostatin-1 | [1] |
| 2. | 5287969 | Flavopiridol | [7] |
| 3. | 5330286 | PD0332991 | [8] |
| 4. | 5359405 | Indirubin | [9] |
| 5. | 16747683 | AZD5438 | [10] |
| 6. | CCT020312 | CCT020312 | [2] |
| 7. | 3071 | 3,3'-diindolylmethane | [3] |
| 8. | 30323 | Daunorubicin |  |
| 9. | 31703 | Doxorubicin |  |
| 10. | 34698 | Teniposide | [4,5] |
| 11. | 88881 | 5,7-dimethoxyflavone | [6] |
| 12. | 119182 | Clofarabine |  |
| 13. | 126941 | Methotrexate |  |
| 14. | 248862 | Nelarabine |  |
| 15. | 667490 | 6-Mercaptopurine | [11] |
| 16. | 969516 | Curcumin | [12] |
| 17. | 1548910 | cis-Resveratrol | [13] |
| 18. | 160355 | R-Roscovitine | [56] |
| 19. | 36314 | Paclitaxel |  |
| 20. | 40839 | Vindesine |  |

*The drugs without references were retrieved from [http://www.cancer.gov/cancertopics/druginfo/ leukemia#dal1](http://www.cancer.gov/cancertopics/druginfo/ leukemia" \l "dal1); [www.drugbank.ca](http://www.drugbank.ca/)

**Supplementary Information:**

**References:**

1. Ardekani AM, Fard SS, Jeddi-Tehrani M, Ghahremanzade R (2011) Bryostatin-1, Fenretinide and 1α,25 (OH)(2)D(3) Induce Growth Inhibition, Apoptosis and Differentiation in T and B Cell-Derived Acute Lymphoblastic Leukemia Cell Lines (CCRF-CEM and Nalm-6). Avicenna J Med Biotechnol 3:177-1793.
2. Stockwell SR, Platt G, Barrie SE, Zoumpoulidou G, Te Poele RH, et al. (2012) Mechanism-based screen for G1/S checkpoint activators identifies a selective activator of EIF2AK3/PERK signalling. PLoS One 7:e28568.
3. Shorey LE, Hagman AM, Williams DE, Ho E, Dashwood RH, Benninghoff AD (2012) 3,3'-Diindolylmethane induces G1 arrest and apoptosis in human acute T-cell lymphoblastic leukemia cells. PLoS One 7:e34975.
4. Rivera GK, Evans WE (1992). Clinical trials of teniposide (VM-26) in childhood acute lymphocytic leukemia. Semin Oncol 19:51-8.
5. Joyce MJ, Pollock BH, Devidas M, Buchanan GR, Camitta B (2013) Chemotherapy for initial induction failures in childhood acute lymphoblastic leukemia: a Children's Oncology Group Study (POG 8764). J Pediatr Hematol Oncol 35:32-35.
6. Goto H, Yanagimachi M, Goto S, Takeuchi M, Kato H, et al. (2012) Methylated chrysin reduced cell proliferation, but antagonized cytotoxicity of other anticancer drugs in acute lymphoblastic leukemia. Anticancer Drugs 23:417-425.
7. Karp JE, Smith BD, Resar LS, Greer JM, Blackford A, et al. (2011) Phase 1 and pharmacokinetic study of bolus-infusion flavopiridol followed by cytosine arabinoside and mitoxantrone for acute leukemias. Blood 117:3302-3310.
8. Kuo TC, Chavarria-Smith JE, Huang D, Schlissel MS (2011) Forced expression of cyclin-dependent kinase 6 confers resistance of pro-B acute lymphocytic leukemia to Gleevec treatment. Mol Cell Biol 31:2566-2576.
9. Kim SH, Choi SJ, Kim YC, Kuh HJ (2009) Anti-tumor activity of noble indirubin derivatives in human solid tumor models in vitro. Arch Pharm Res 32:915-922.
10. Byth KF, Thomas A, Hughes G, Forder C, McGregor A, et al. (2009) AZD5438, a potent oral inhibitor of cyclin-dependent kinases 1, 2, and 9, leads to pharmacodynamic changes and potent antitumor effects in human tumor xenografts. Mol Cancer Ther 8:1856-1866.
11. Bostrom B, Erdmann G (1993) Cellular pharmacology of 6-mercaptopurine in acute lymphoblastic leukemia. Am J Pediatr Hematol Oncol 15:80-6.
12. Aggarwal BB, Banerjee S, Bharadwaj U, Sung B, Shishodia S, Sethi G (2007) Curcumin induces the degradation of cyclin E expression through ubiquitin-dependent pathway and up-regulates cyclin-dependent kinase inhibitors p21 and p27 in multiple human tumor cell lines. Biochem Pharmacol 73:1024-1032.
13. Zunino SJ, Storms DH (2006) Resveratrol-induced apoptosis is enhanced in acute lymphoblastic leukemia cells by modulation of the mitochondrial permeability transition pore. Cancer Lett 240:123-134.
14. Yeh RD, Chen JC, Lai TY, Yang JS, Yu CS, et al. (2011) Gallic acid induces G₀/G₁ phase arrest and apoptosis in human leukemia HL-60 cells through inhibiting cyclin D and E, and activating mitochondria-dependent pathway. Anticancer Res 31:2821-2832.
15. Mantena SK, Sharma SD, Katiyar SK (2006) Berberine inhibits growth, induces G1 arrest and apoptosis in human epidermoid carcinoma A431 cells by regulating Cdki-Cdk-cyclin cascade, disruption of mitochondrial membrane potential and cleavage of caspase 3 and PARP. Carcinogenesis 27:2018-2027.
16. Yu CS, Huang AC, Yang JS, Yu CC, Lin CC, et al. (2012) Safrole induces G0/G1 phase arrest via inhibition of cyclin E and provokes apoptosis through endoplasmic reticulum stress and mitochondrion-dependent pathways in human leukemia HL-60 cells. Anticancer Res 32:1671-1679.
17. Adhami VM, Aziz MH, Reagan-Shaw SR, Nihal M, Mukhtar H, Ahmad N (2004) Sanguinarine causes cell cycle blockade and apoptosis of human prostate carcinoma cells via modulation of cyclin kinase inhibitor-cyclin-cyclin-dependent kinase machinery. Mol Cancer Ther 3:933-940.
18. Gong J, Traganos F, Darzynkiewicz Z (1994) Use of the cyclin e restriction point to map cell arrest in g(1)-induced by N-butyrate, cycloheximide, staurosporine, lovastatin, mimosine and quercetin. Int J Oncol 4:803-808.
19. Schnier JB, Nishi K, Goodrich DW, Bradbury EM (1996) G1 arrest and down-regulation of cyclin E/cyclin-dependent kinase 2 by the protein kinase inhibitor staurosporine are dependent on the retinoblastoma protein in the bladder carcinoma cell line 5637. Proc Natl Acad Sci USA 93:5941-5946.
20. Relja B, Meder F, Wilhelm K, Henrich D, Marzi I, Lehnert M (2010) Simvastatin inhibits cell growth and induces apoptosis and G0/G1 cell cycle arrest in hepatic cancer cells. Int J Mol Med 26:735-741.
21. Torricelli P, Caraglia M, Abbruzzese A, Beninati S (2013) γ-Tocopherol inhibits human prostate cancer cell proliferation by up-regulation of transglutaminase 2 and down-regulation of cyclins. Amino Acids 44:45-51.
22. Chen CH, Huang TS, Wong CH, Hong CL, Tsai YH, et al. (2009) Synergistic anti-cancer effect of baicalein and silymarin on human hepatoma HepG2 Cells. Food Chem Toxicol 47:638-644.
23. Yim D, Singh RP, Agarwal C, Lee S, Chi H, Agarwal R (2005) A novel anticancer agent, decursin, induces G1 arrest and apoptosis in human prostate carcinoma cells. Cancer Res 65:1035-1044.
24. Singh RP, Agrawal P, Yim D, Agarwal C, Agarwal R (2005) Acacetin inhibits cell growth and cell cycle progression, and induces apoptosis in human prostate cancer cells: structure-activity relationship with linarin and linarin acetate. Carcinogenesis 26:845-854.
25. Khuntawee W, Rungrotmongkol T, Hannongbua S (2012) Molecular dynamic behavior and binding affinity of flavonoid analogues to the cyclin dependent kinase 6/cyclin D complex. J Chem Inf Model 52:76-83.
26. Lu H, Chang DJ, Baratte B, Meijer L, Schulze-Gahmen U (2005) Crystal structure of a human cyclin-dependent kinase 6 complex with a flavonol inhibitor, fisetin. J Med Chem 48:737-743.
27. Lu H, Schulze-Gahmen U (2006) Toward understanding the structural basis of cyclin-dependent kinase 6 specific inhibition. J Med Chem 49:3826-3831.
28. Kawanishi N, Sugimoto T, Shibata J, Nakamura K, Masutani K, et al. (2006) Structure-based drug design of a highly potent CDK1,2,4,6 inhibitor with novel macrocyclic quinoxalin-2-one structure. Bioorg Med Chem Lett 16:5122-5126.
29. Sun Y, Cai TT, Zhou XB, Xu Q (2009) Saikosaponin a inhibits the proliferation and activation of T cells through cell cycle arrest and induction of apoptosis. Int Immunopharmacol 9:978-983.
30. Cho YS, Borland M, Brain C, Chen CH, Cheng H, et al. (2010) 4-(Pyrazol-4-yl)-pyrimidines as selective inhibitors of cyclin-dependent kinase 4/6. J Med Chem 53:7938-7957.
31. Zi X, Agarwal R (1999) Silibinin decreases prostate-specific antigen with cell growth inhibition via G1 arrest, leading to differentiation of prostate carcinoma cells: implications for prostate cancer intervention. Proc Natl Acad Sci USA 96:7490-7495.
32. Mao X, Liang SB, Hurren R, Gronda M, Chow S, et al. (2008) Cyproheptadine displays preclinical activity in myeloma and leukemia. Blood 112:760-769.
33. Chen Z, Wu Q, Chen Y, He J (2008) Effects of betulinic acid on proliferation and apoptosis in Jurkat cells and its in vitro mechanism. J Huazhong Univ Sci Technolog Med Sci 28:634-638.
34. Mao X, Cao B, Wood TE, Hurren R, Tong J, et al. (2011) A small-molecule inhibitor of D-cyclin transactivation displays preclinical efficacy in myeloma and leukemia via phosphoinositide 3-kinase pathway. Blood 117:1986-1997.
35. Lee KW, Kim HJ, Lee YS, Park HJ, Choi JW, et al. (2007) Acteoside inhibits human promyelocytic HL-60 leukemia cell proliferation via inducing cell cycle arrest at G0/G1 phase and differentiation into monocyte. Carcinogenesis 28:1928-1936.
36. Schult C, Dahlhaus M, Glass A, Fischer K, Lange S, et al. (2012) The dual kinase inhibitor NVP-BEZ235 in combination with cytotoxic drugs exerts anti-proliferative activity towards acute lymphoblastic leukemia cells. Anticancer Res 32:463-474.
37. Hleb M, Murphy S, Wagner EF, Hanna NN, Sharma N, et al. (2004) Evidence for cyclin D3 as a novel target of rapamycin in human T lymphocytes. J Biol Chem 279:31948-31955.
38. Nair HK, Rao KV, Aalinkeel R, Mahajan S, Chawda R, Schwartz SA (2004) Inhibition of prostate cancer cell colony formation by the flavonoid quercetin correlates with modulation of specific regulatory genes. Clin Diagn Lab Immunol 11:63-69.
39. Lu D, Liu JX, Endo T, Zhou H, Yao S, et al. (2009) Ethacrynic acid exhibits selective toxicity to chronic lymphocytic leukemia cells by inhibition of the Wnt/beta-catenin pathway. PLoS One 4:e8294.
40. Gamazon ER, Huang RS, Dolan ME, Cox NJ (2011) Copy number polymorphisms and anticancer pharmacogenomics. Genome Biol 12:R46.
41. Phromnoi K, Reuter S, Sung B, Limtrakul P, Aggarwal BB (2010) A Dihydroxy-pentamethoxyflavone from Gardenia obtusifolia suppresses proliferation and promotes apoptosis of tumor cells through modulation of multiple cell signaling pathways. Anticancer Res 30:3599-3610.
42. Villerbu N, Gaben AM, Redeuilh G, Mester J (2002) Cellular effects of purvalanol A: a specific inhibitor of cyclin-dependent kinase activities. Int J Cancer 97:761-769.
43. Jiménez-Orozco FA, López-González JS, Nieto-Rodriguez A, Velasco-Velázquez MA, Molina-Guarneros JA, et al. (2001) Decrease of cyclin D1 in the human lung adenocarcinoma cell line A-427 by 7-hydroxycoumarin. Lung Cancer 34:185-194.
44. Choi EJ, Kim GH (2008) Daidzein causes cell cycle arrest at the G1 and G2/M phases in human breast cancer MCF-7 and MDA-MB-453 cells. Phytomedicine 15:683-690.
45. Sanchez-Martinez C, Shih C, Faul MM, Zhu G, Paal M, et al. (2003) Aryl[a]pyrrolo[3,4-c]carbazoles as selective cyclin D1-CDK4 inhibitors. Bioorg Med Chem Lett 13:3835-3839.
46. Parry D, Guzi T, Shanahan F, Davis N, Prabhavalkar D, et al. (2010) Dinaciclib (SCH 727965), a novel and potent cyclin-dependent kinase inhibitor. Mol Cancer Ther 9:2344-2353.
47. Clare PM, Poorman RA, Kelley LC, Watenpaugh KD, Bannow CA, Leach KL (2001) The cyclin-dependent kinases cdk2 and cdk5 act by a random, anticooperative kinetic mechanism. J Biol Chem 276:48292-48299.
48. Simone M, Erba E, Damia G, Vikhanskaya F, Di Francesco AM, et al. (2005) Variolin B and its derivate deoxy-variolin B: new marine natural compounds with cyclin-dependent kinase inhibitor activity. Eur J Cancer 41:2366-2377.
49. Bettayeb K, Tirado OM, Marionneau-Lambot S, Ferandin Y, Lozach O, et al. (2007) Meriolins, a new class of cell death inducing kinase inhibitors with enhanced selectivity for cyclin-dependent kinases. Cancer Res 67:8325-8334.
50. Lee J, Kim KH, Jeong S (2011) Discovery of a novel class of 2-aminopyrimidines as CDK1 and CDK2 inhibitors. Bioorg Med Chem Lett 21:4203-4205.
51. Martin MP, Alam R, Betzi S, Ingles DJ, Zhu JY, Schönbrunn E (2012) A novel approach to the discovery of small-molecule ligands of CDK2. Chembiochem 13:2128-2136.
52. Alessi F, Quarta S, Savio M, Riva F, Rossi L, (1998). The cyclin-dependent kinase inhibitors olomoucine and roscovitine arrest human fibroblasts in G1 phase by specific inhibition of CDK2 kinase activity. Exp Cell Res 245:8-18.
53. Heathcote DA, Patel H, Kroll SH, Hazel P, Periyasamy M, et al. (2010) A novel pyrazolo[1,5-a]pyrimidine is a potent inhibitor of cyclin-dependent protein kinases 1, 2, and 9, which demonstrates antitumor effects in human tumor xenografts following oral administration. J Med Chem 53:8508-8522.
54. de Bruijn P, Moghaddam-Helmantel IM, de Jonge MJ, Meyer T, Lam MH, et al. (2009) Validated bioanalytical method for the quantification of RGB-286638, a novel multi-targeted protein kinase inhibitor, in human plasma and urine by liquid chromatography/tandem triple-quadrupole mass spectrometry. J Pharm Biomed Anal 50:977-982.
55. Sugiyama K, Akiyama T, Shimizu M, Tamaoki T, Courage C, et al. (1999) Decrease in susceptibility toward induction of apoptosis and alteration in G1 checkpoint function as determinants of resistance of human lung cancer cells against the antisignaling drug UCN-01 (7-Hydroxystaurosporine). Cancer Res 59:4406-4412.
56. Federico M, Symonds CE, Bagella L, Rizzolio F, Fanale D, et al. (2010) R-Roscovitine (Seliciclib) prevents DNA damage-induced cyclin A1 upregulation and hinders non-homologous end-joining (NHEJ) DNA repair. Mol Cancer 9:208.

**Table S2. Hydrogen bond distances**

|  | **Drug** | **Element(Atom-H)** | **Element(Atom-A)** | **Distance (< 2.500)** |
| --- | --- | --- | --- | --- |
| **CCNE1** |  |  |  |  |
| 1 | Doxorubicin | H(2506)Asn 236 | O(4448) | 1.979 |
|  |  | H(4500) | O(1719)Glu188 | 2.077 |
|  |  | H(4502) | O(2724)Val 250 | 1.874 |
| 2 | Daunorubicin | H(2506)Asn 236 | O(4448) | 1.922 |
|  |  | H(4496) | O(1719)Glu 188 | 1.641 |
|  |  | H(4500) | O(1719)Glu 188 | 1.614 |
|  |  | H(4502) | O(2724)Val 250 | 1.933 |
| 3 | Baicalein | H(4471) | O(2740)Leu251 | 2.042 |
| 4 | Teniposide | H(359)Lys 108 | O(4444) | 2.274 |
| 5 | Flavopiridol | H(4485) | O(2724)Val 250 | 1.843 |
| 6 | CCT020312 | H(4488) | O(2724)Val 250 | 2.28 |
|  |  | H(4511) | O(4176)Asp 341 | 1.726 |
| 7 | Silymarin | H(125)Trp 95 | O(4449) | 2.288 |
|  |  | H(4489) | O(2740)Leu 251 | 1.86 |
| 8 | Chrysin | H(4470) | O(2740)Leu 251 | 2.049 |
| 9 | Simvastatin | H(2506)Asn 236 | O(4446) | 2.035 |
|  |  | H(4506) | O(2740)Leu 251 | 2.018 |
| 10 | AZD5438 | H(2506)Asn 236 | O(4444) | 2.239 |
|  |  | H(4480) | O(2724)Val 250 | 1.88 |
| 11 | Nelarabine | H(2506)Asn 236 | O(4443) | 2.349 |
|  |  | H(4470) | O(2740)Leu 251 | 2.052 |
|  |  | H(4472) | O(2740)Leu 251 | 1.849 |
| 12 | Methotrexate | H(359)Lys 108 | O(4442) | 1.974 |
|  |  | H(125)Trp 95 | O(4445) | 1.881 |
| 13 | 5,7-Dimethoxyflavone | H(2826)Tyr255 | O(4444) | 2.406 |
| 14 | PD0332991 | H(125)Trp95 | O(4443) | 2.051 |
|  |  | H(4504) | O(4176)Asp 341 | 1.893 |
| 15 | Berberine | H(2826)Tyr255 | O(4443) | 2.199 |
| 16 | Curcumin | H(2506)Asn 236 | O(4447) | 2.331 |
|  |  | H(4482) | O(1719)Glu 188 | 1.642 |
| 17 | Clofarabine | H(2506)Asn 236 | O(4446) | 2.411 |
|  |  | H(4469) | O(2740)Leu 251 | 2.044 |
| 18 | cis-Resveratrol | H(4468) | O(2740)Leu 251 | 2.023 |
| 19 | Gamma-Tocopherol | H(360)Lys 108 | O(4443) | 2.366 |
|  |  | H(4519) | O(2360)Pro 228 | 2.227 |
| 20 | Sanguinarine | H(4221)Asn 344 | O(4443) | 1.819 |
| 21 | Staurosporine | H(4491) | O(2724)Val 250 | 1.854 |
| 22 | Bryostatin-1 | H(2506)Asn 236 | O(4453) | 2.065 |
|  |  | H(4523) | O(1719)Glu 188 | 2.252 |
| 23 | Gallic acid | H(360)Lys 108 | O(4445) | 2.022 |
|  |  | H(4456) | O(1718)Glu 188 | 1.725 |
|  |  | H(4457) | O(1718)Glu 188 | 1.663 |
|  |  | H(4458) | O(2351)Ser 227 | 2.133 |
| 24 | 6-Mercaptopurine | H(4454) | O(2724)Val 250 | 2.098 |
| **CCND3** |  |  |  |  |
| 1 | Nelarabine | H(3696) | O(264)Tyr 38 | 2.327 |
|  |  | H(3697) | O(2910)Tyr 198 | 1.825 |
|  |  | H(3699) | O(1086)Asp 86 | 1.693 |
| 2 | Clofarabine | H(3696) | O(2733)Leu 186 | 1.707 |
|  |  | H(3698) | O(1086)Asp 86 | 1.781 |
| 3 | 6-Mercaptopurine | H(2241)Ala 157 | N(3671) | 2.268 |
|  |  | H(1038)Asn 83 | N(3673) | 2.122 |
| 4 | cis-Resveratrol | H(3695) | O(1086)Asp 86 | 1.809 |
| **CCND1** |  |  |  |  |
| 1 | Curcumin | H(3937) | O(1733)Ser 131 | 2.117 |
|  |  | H(3938) | O(1251)Gln 100 | 1.837 |
| 2 | Baicalein | H(3925) | O(1171)Lys 96 | 1.974 |
| 3 | 7-hydroxycoumarin | H(3915) | O(1171) | 1.874 |
| 4 | PurvalanolA | H(3944) | O(1251)Gln 100 | 2.298 |
|  |  | H(3945) | O(1171)Lys 98 | 1.884 |
| 5 | Arcyriaflavin | H(3923) | O(1251)Gln 100 | 1.984 |
| 6 | Ethacrynic acid | H(1253)Gln 100 | O(3902) | 2.02 |
|  |  | H(1260)Gln 100 | O(3903) | 2.099 |
| **CCNA1** |  |  |  |  |
| 1 | Daunorubicin | H(4273) | O(783)Glu 269 | 2.412 |
|  |  | H(4282) | O(778)Glu 268 | 1.843 |
|  |  | H(4284) | O(754)Lys 266 | 1.991 |
| 2 | PD0332991 | H(4286) | O(17)Glu 174 | 1.714 |
| 3 | Flavopiridol | H(4263) | O(754)Lys 266 | 2.067 |
|  |  | H(4272) | O(754)Lys 266 | 1.457 |
| 4 | Doxorubicin | H(4274) | O(763)Phe 267 | 1.902 |
|  |  | H(4278) | O(778)Glu 268 | 1.535 |
|  |  | H(4282) | O(779)Glu 268 | 2.458 |
|  |  | H(4284) | O(754)Lys 266 | 1.956 |
|  |  | H(4286) | O(792)Ile 270 | 2.108 |
| 5 | R-Roscovitine | H(3220)Ala 307 | O(4224) | 2.099 |
|  |  | H(4254) | O(1077)Phe 304 | 2.133 |
|  |  | H(4270) | O(1077)Phe 304 | 2.279 |
| 6 | cis-Resveratrol | H(4250) | O(735)Leu 263 | 2.041 |
|  |  | H(4252) | O(1077)Phe 304 | 1.893 |
| 7 | 3,3'-Diindolylmethane | H(4247) | O(1077)Phe 304 | 1.789 |
| 8 | Clofarabine | H(4251) | O(779)Glu 268 | 1.971 |
|  |  | H(4252) | O(778)Glu 268 | 2.071 |
|  |  | H(4253) | O(1104)Ala 307 | 2.01 |
| 9 | Curcumin | H(4264) | O(4)Asn 173 | 1.987 |
| 10 | Nelarabine | H(4251) | O(486)Glu 230 |  |
| 11 | Methotrexate | H(2568)Asn 229 | O(4227) | 2.144 |
|  |  | H(3257)Asn 312 | O(4228) | 1.771 |
|  |  | H(2108)Asn 173 | N(4233) | 2.085 |
|  |  | H(4276) | O(7)Asn 173 | 1.862 |
| 12 | CCT020312 | H(4270) | O(1147)Gln 313 | 2.199 |
|  |  | H(4293) | O(779)Glu 268 | 2.035 |
| 13 | AZD5438 | H(4262) | O(778)Glu 268 | 1.945 |
| 14 | 6-Mercaptopurine | H(4237) | O(754)Lys 266 | 2.044 |
| **CDK6** |  |  |  |  |
|  | 3NU | H(1137)Val 101 | N(4092) | 2.228 |
|  |  | H(4111) | O(1133)Val 101 | 1.794 |
| 1 | CHEMBL1230169 | H(1137)Val 101 | N(4090) | 2.155 |
|  |  | H(4118) | O(1133)Val 101 | 1.901 |
| 2 | Doxorubicin | H(1137)Val 101 | O(4096) | 2.36 |
|  |  | H(4141) | O(1182)Asp 104 | 1.998 |
|  |  | H(4147) | O(1133)Val 101 | 1.781 |
|  |  | H(4149) | O(1149)Asp 102 | 2.008 |
| 3 | Indirubin | H(1137)Val 101 | O(4088) | 2.034 |
|  |  | H(4108) | O(1101)Glu 99 | 2.101 |
| 4 | Daunorubicin | H(1137)Val 101 | O(4095) | 2.084 |
|  |  | H(4140) | O(1182)Asp 104 | 1.682 |
|  |  | H(4147) | O(1133)Val 101 | 1.645 |
| 5 | Fisetin | H(4114) | O(1133)Val 101 | 2.358 |
|  |  | H(4116) | O(1182)Asp 104 | 2.394 |
|  |  |  |  |  |
| 6 | Curcumin | H(446)Lys 43 | O(4089) | 2.287 |
|  |  | H(2142)Asp 163 | O(4089) | 2.041 |
|  |  | H(1137) | O(4091)Val 101 | 2.004 |
|  |  | H(4127) | O(2140)Asp 163 | 1.796 |
| 7 | Saikosaponin A | H(4176) | O(1859)Asp 145 | 2.115 |
|  |  | H(4188) | O(2140)Asp 163 | 2.013 |
|  |  | H(4204) | O(1133)Val 101 | 1.886 |
|  |  | H(4207) | O(1133)Val 101 | 2.363 |
|  |  | H(4208) | O(1182)Asp 104 | 2.462 |
| 8 | Flavopiridol | H(1137) Val 101 | O(4092) | 2.069 |
|  |  | H(4126) | O(1922)Gln 149 | 1.811 |
| 9 | AZD5438 | H(4125) | O(1133)Val 101 | 2.059 |
| 10 | 3,3'-diindolymethane | H(4110) | N(1122)His 100 | 2.16 |
|  |  | H(4111) | O(1101)Glu 99 | 2.164 |
| 11 | PD0332991 | H(446)Lys 43 | O(4088) | 2.057 |
|  |  | H(4141) | O(1225)Thr 107 | 1.852 |
|  |  | H(4142) | O(1133)Val 101 | 2.011 |
| 12 | Apigenin | H(4114) | O(1133)Val 101 | 1.907 |
|  |  | H(4115) | O(1182)Asp 104 | 1.708 |
| 13 | Silibinin | H(1137)Val 101 | O(4091) | 1.885 |
|  |  | H(4134) | O(1225)Thr 107 | 1.736 |
|  |  | H(4139) | O(110)Ile 19 | 1.926 |
| 14 | Acacetin | H(446)Lys 43 | O(4091) | 2.348 |
|  |  | H(4115) | O(1133)Val 101 | 1.807 |
|  |  | H(4116) | O(1182)Asp 104 | 1.852 |
| 15 | Clofarabine | H(4114) | O(1922)Gln 149 | 1.918 |
|  |  | H(4115) | O(1922)Gln 149 | 2.22 |
|  |  | H(4116) | O(1101)Glu 99 | 1.951 |
| 16 | Chrysin | H(4114) | O(1133) Val 101 | 1.922 |
|  |  | H(4115) | O(1182)Asp 104 | 1.624 |
| 17 | Aminopurvalanol | H(4134) | O(1133)Val 101 | 2.485 |
|  |  | H(4135) | O(1922)Gln 149 | 1.912 |
|  |  | H(4140) | N(1122)His 100 | 2.209 |
| 18 | cis-Resveratrol | H(4114) | O(1922)Gln 149 | 1.824 |
|  |  | H(4115) | O(1101)Glu 99 | 1.836 |
| 19 | CCT020312 | H(4133) | O(1133)Val 101 | 1.945 |
| 20 | 5,7-dimethoxyflavone | H(1137)Val 101 | O(4090) | 2.03 |
| 21 | 6-Mercaptopurine | H(1137)Val 101 | N(4089) | 2.3 |
|  |  | H(4100) | O(1101)Glu 99 | 1.865 |
| 22 | Decursin | H(446)Lys 43 | O(4091) | 2.433 |
|  |  | H(2142)Asp 163 | O(4091) | 2.101 |
| 23 | Nelarabine | H(4114) | O(1922)Gln 149 | 2.216 |
|  |  | H(4117) | O(1133)Val 101 | 1.894 |
| 24 | Paclitaxel | H(1902)Lys 147 | O(4090) | 1.934 |
|  |  | H(139)Gly 25 | O(4095) | 1.844 |
|  |  | H(4187) | O(2140)Asp 163 | 1.848 |
| 25 | Methotrexate | H(446)Lys 43 | O(4091) | 2.25 |
|  |  | H(4136) | O(1182)Asp 104 | 2.287 |
| 26 | Vindesine | H(4165) | O(95) Glu 18 | 2.119 |
|  |  | H(4186) | O(137) Gly 25 | 2.11 |
| 27 | Teniposide | H(139)Gly 25 | O(4089) | 2.396 |
| **CDK2** |  |  |  |  |
| 1 | Doxorubicin | H(4898) | O(2386)Asp 145 | 1.955 |
|  |  | H(4904) | O(175)Ile 10 | 1.958 |
|  |  | H(4906) | O(2185)Asn 132 | 2.24 |
| 2 | Daunorubicin | H(4904) | O(175)Ile 10 | 1.875 |
|  |  | H(4911) | O(2387)Asp 145 | 1.729 |
| 3 | Acetoside | H(2145)Lys 129 | O(4844) | 2.482 |
|  |  | H(4903) | O(2102)Asp 127 | 2.097 |
|  |  | H(4905) | O(2387)Asp 145 | 1.709 |
|  |  | H(4906) | O(2185)Asn 132 | 2.214 |
|  |  | H(4919) | O(2651)Glu 162 | 1.924 |
|  |  | H(4922) | O(821)Glu 51 | 1.637 |
| 4 | Flavopiridol | H(4886) | O(1426)Asp 86 | 2.122 |
|  |  | H(4887) | O(1369)Leu 83 | 2.044 |
| 5 | Curcumin | H(207)Glu 12 | O(4848) | 2.14 |
|  |  | H(4884) | O(1369)Leu 83 | 1.999 |
| 6 | Quercetin | H(2388)Asp 145 | O(4848) | 2.438 |
|  |  | H(4873) | O(822)Glu 51 | 2.109 |
|  |  | H(4874) | O(2165)Gln 131 | 2.134 |
| 7 | Clofarabine | H(4872) | O(2386)Asp 145 | 1.684 |
| 8 | CHEMBL1234833 | H(4892) | O(2386)Asp 145 | 1.728 |
| 9 | SCH72965_Dinaclib | H(230)Thr 14 | O(4845) | 2.055 |
|  |  | H(4887) | O(258)Gly 16 | 1.777 |
| 10 | cis-Resveratrol | H(4870) | O(821)Glu 51 | 1.769 |
|  |  | H(4871) | O(752)Thr 47 | 1.869 |
|  |  | H(4872) | O(2387)Asp 145 | 1.684 |
| 11 | Roscovitine | H(4884) | O(752)Thr 47 | 1.898 |
|  |  | H(4890) | O(741)Ser 46 | 1.948 |
| 12 | Nelarabine | H(4871) | O(2387)Asp 145 | 1.838 |
|  |  | H(4874) | O(752)Thr 47 | 1.996 |
| 13 | Methotrexate | H(544)Lys 33 | O(4844) | 1.622 |
|  |  | H(246)Tyr 15 | O(4847) | 2.195 |
|  |  | H(227)Thr 14 | O(4848) | 2.034 |
|  |  | H(4894) | O(1369)Leu 83 | 1.919 |
| 14 | PD0332991 | H(4899) | O(2387)Asp 145 | 2.175 |
| 15 | Indirubin | H(246)Tyr 15 | O(4844) | 2.08 |
|  |  | H(227)Thr 14 | O(4845) | 2.13 |
| 16 | CCT020312 | H(2145)Lys 129 | O(4886) | 2.141 |
| 17 | 3,3'-Diindolymethane | H(4868) | O(752)Thr 47 | 2.075 |
| 18 | AZD5438 | H(4882) | O(752)Thr 47 | 2.005 |
| 19 | Teniposide | H(2145)Lys 129 | O(4846) | 2.288 |
| 20 | Chrysin | H(246)Tyr 15 | O(4846) | 2.268 |
|  |  | H(4872) | O(821)Glu 51 | 1.628 |
| 21 | OSO | H(4872) | O(2185)Asn 132 | 1.995 |
|  |  | H(4873) | O(2165)Gln 131 | 2.102 |
| 22 | Meriolin3 | H(544)Lys 33 | N(4847) | 2.447 |
|  |  | H(4863) | O(752)Thr 47 | 1.893 |
| 23 | RGB286638 | H(2388)Asp 145 | O(4844) | 2.042 |
|  |  | H(4910) | O(752)Thr 47 | 2.379 |
| 24 | 5,7-Dimethoxyflavone | H(227)Thr 14 | O(4847) | 2.073 |
| 25 | U55 | H(4868) | O(821)Glu 51 | 1.771 |
|  |  | H(4871) | O(2387)Asp 145 | 1.694 |
|  |  | H(4872) | O(2102)Asp 127 | 1.893 |
| 26 | Variolin B | H(4876) | O(821)Glu 51 | 1.749 |
| 27 | 6-Mercaptopurine | H(4857) | O(752)Thr 47 | 2.288 |
